# When Dielectric Constants Deceive: Interrogating Solvation in Ionic Liquids with Cyclic Voltammetry

**DOI:** 10.1021/acs.jpcb.6c00284

**Published:** 2026-02-26

**Authors:** Johannes Wega, Franck Guignard, Eric Vauthey

**Affiliations:** Department of Physical Chemistry, University of Geneva, Quai Ernest Ansermet 30, Geneva CH-1211, Switzerland

## Abstract

Ionic liquids are increasingly discussed as alternatives to conventional organic solvents for applications based on photoinduced electron transfer. For the rational design of such applications, reliable estimates of electron-transfer driving forces are essential. Based on the Born model of solvation, the moderate dielectric constants of ionic liquids (ε_r_ ≈ 8 – 15) suggest that they should resemble medium-polarity solvents such as dichloromethane or pyridine in photoinduced electron transfer and exhibit comparable solvation energies. Here, we test this assumption by experimentally comparing the solvation energies of three small organic solutes relevant to photochemistry in several imidazolium-based ionic liquids and in conventional dipolar solvents. Solvation energies were inferred from shifts of half-wave reduction potentials obtained from cyclic voltammetry. We find that, for the investigated solutes, ionic liquids provide solvation energies comparable to those of strongly polar solvents such as acetonitrile or dimethyl sulfoxide. While the organic solvents follow the qualitative trend predicted by the Born equation, ionic liquids deviate from it and yield much larger solvation energies compared to dipolar solvents of the same dielectric constant. This behavior is attributed to the intrinsically high ionic strength of ionic liquids, which enhances electrostatic screening and results in substantially larger solvation energies and consequently much larger driving forces for photoinduced electron transfer than would be expected based on their dielectric constants alone.

## Graphical abstract

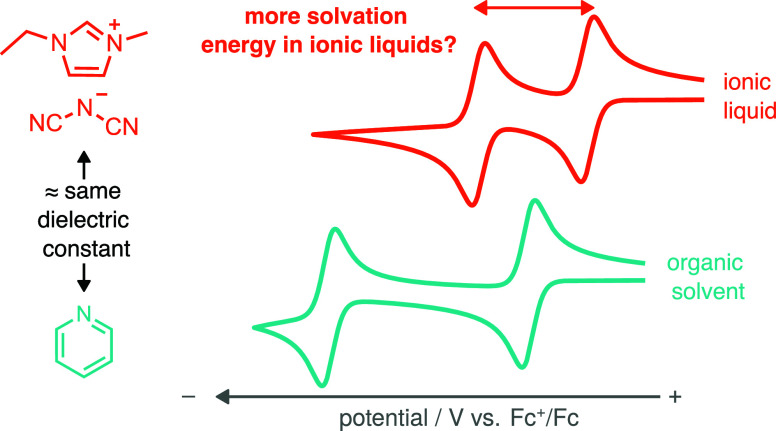

## 1 Introduction

Photoinduced electron transfer lies at the heart of many light-driven technologies such as photoredox catalysis,^1,2^ organic photovoltaics,^3,4^ and artificial photosynthesis.^5–7^ For the rational design of such applications, it is essential to estimate and tune the driving force of the initial charge-separation reaction (Δ*G*_CS_^0^). In liquid solution, the driving force for a photoinduced electron transfer reaction, for example when the donor (D) is photoexcited:

$$\begin{array}{l} D\overset{h\nu }{\to }D^{*}\\ D^{*}+A\to D^{•+}+A^{•-} \end{array}$$ 1

can be estimated using the Weller equation^8^:

$$\Delta G_{\mathrm{CS}}^{0}=F\left[E_{D^{•+}/D}^{0}-E_{A/A^{•-}}^{0}\right]-E^{*}+U\left(R\right)$$ 2

Here, *F* is Faraday’s constant, E* is the excited-state energy of D, and *U*(*R*) denotes the electrostatic attraction between the ions D^•+^ and A^•–^ at a distance *R*. The terms *E*_D^•+^/D_^0^ and *E*_A/A^•–^_^0^ are the electrochemical standard reduction potentials of D^•+^ and A respectively. The equation is equally valid if the acceptor (A) is photoexcited, in which case E* corresponds to the excited-state energy of A.

It is important to note that, in order to obtain an accurate value of Δ*G*_CS_^0^, the reduction potentials *E* ^0^ must be taken from measurements performed in the same solvent in which the driving force is to be evaluated. This is because electrochemical reduction potentials include the solvation energies (vide infra) of the species involved, which depend on the solvent used in the measurement. Oftentimes, experimental electrochemical reduction potentials are only available in polar solvents such as acetonitrile. If one wishes to estimate the driving force of a photoinduced electron transfer reaction in a different solvent, a correction must be applied to the Weller equation, as the reduction potentials contain solvation energies specific to acetonitrile rather than to the solvent/medium of interest.

A crude correction, known as the Born correction,^9–11^ can be applied by subtracting the solvation energies of the ionic species in reaction 1 in the solvent in which the reduction potentials were measured (with dielectric constant ε_r,ref_) from the Weller equation, and adding instead the corresponding solvation energies in the target solvent with dielectric constant ε_r,tar_. In this correction, the solvation energies of the ionic species are calculated using the Born equation, which treats the molecular ions as spheres of radius *r*. The solvation energy is then obtained as the electrostatic work *W* required to charge up a sphere in vacuum (ε_r_ = 1) with charge *z* · *e*, relative to the work required to charge the same sphere in a continuous dielectric medium with dielectric constant ε_r_ ^12^:

$$\begin{array}{ll} \Delta G_{\mathrm{solv},\mathrm{Born}}^{0} & =W(\varepsilon_{r})-W(\varepsilon_{r}=1)\\ & =-\frac{z^{2}e^{2}}{8\pi \varepsilon_{0}r}\left(1-\frac{1}{\varepsilon_{r}}\right) \end{array}$$ 3

Thus, the correction term that needs to be added to the Weller equation (eq 2) to calculate the charge separation driving force in a different solvent than the solvent in which the reduction potentials were measured amounts to

$$\mathrm{corr}.=\frac{e^{2}}{8\pi \varepsilon_{0}}\left(\frac{1}{r_{D^{•+}}}+\frac{1}{r_{A^{•-}}}\right)\left(\frac{1}{\varepsilon_{r,\mathrm{tar}}}-\frac{1}{\varepsilon_{r,\mathrm{ref}}}\right)$$ 4

Ionic liquids (ILs) have emerged as an interesting class of materials due to their unique physicochemical properties as essentially molten salts near or at room temperature.^13–21^ They are increasingly discussed as an alternative to conventional organic solvents in chemical synthesis as well as in photoredox catalysis and solar cell applications.^13–15^ Based on their macroscopically measurable dielectric constant, typically on the order of 8–15,^22–24^ one might expect them to behave similarly to moderately polar solvents such as dichloromethane (DCM, ε_r_ = 8.93 ^25^) or pyridine (PYR, ε_r_ = 12.9 ^25^) in electron transfer reactions. On the other hand, the nature of these liquids is fundamentally different from that of conventional solvents, as they consist essentially of a pool of charged ions and hence one might expect a markedly different solvation behavior in these liquids.

The relatively low dielectric constants of ILs would further suggest smaller solvation energies compared to typical polar solvents such as acetonitrile (ACN, ε_r_ = 35.94 ^25^) or dimethyl sulfoxide (DMSO, ε_r_ = 46.45 ^25^) according to the Born equation. This, however, is at odds with solvatochromic measurements,^26–33^ where polar chromophores in ILs exhibit shifts comparable to those measured in ACN or DMSO, pointing to a similar magnitude in solvation energy. Furthermore, photoinduced electron transfer studies^34–36^ show that reactions yielding charged species readily occur in ILs, while the same reactions do not proceed in conventional solvents of similar dielectric constants. This points to significantly higher solvation energies in the ILs, which stabilize the resulting ion-pair state and thereby render the reaction exergonic. These observations are also consistent with empirical solvent polarity scales like the E_T_(30) or Kamlett-Taft π* scales which indicate similar values for ILs and highly polar solvents.^26,37,38^

Recent molecular dynamics simulations by Renfro et al.^39^ point to solvation energies in ILs comparable to those in ACN. The authors attribute this behavior to a distinctly different solvation mechanism resulting from an overscreening effect caused by charge oscillations associated with the structured ion shells of the ionic liquid leading to a strong deviation of the computed solvation energies in ILs from values estimated using the Born equation.^39,40^

However, is the dielectric constant the only bulk property that determines the solvation energy when neglecting any specific solvent–solute interaction? As the name suggests, ILs are essentially molten salts with very high ionic strengths. Even from a basic continuum electrodynamics perspective, this modifies the solvation energy predicted by the Born model. Considering a central anion, for example, counter cations from the IL accumulate around it, and the positive electrostatic potential of this counterion cloud is partially screening the potential of the spherical anion (cf. Figure 1A). As a result, less electrostatic work is required to charge the sphere compared to the case of zero ionic strength, and eq 3 therefore underestimates the magnitude of the ionic solvation energy.

**Figure 1:**
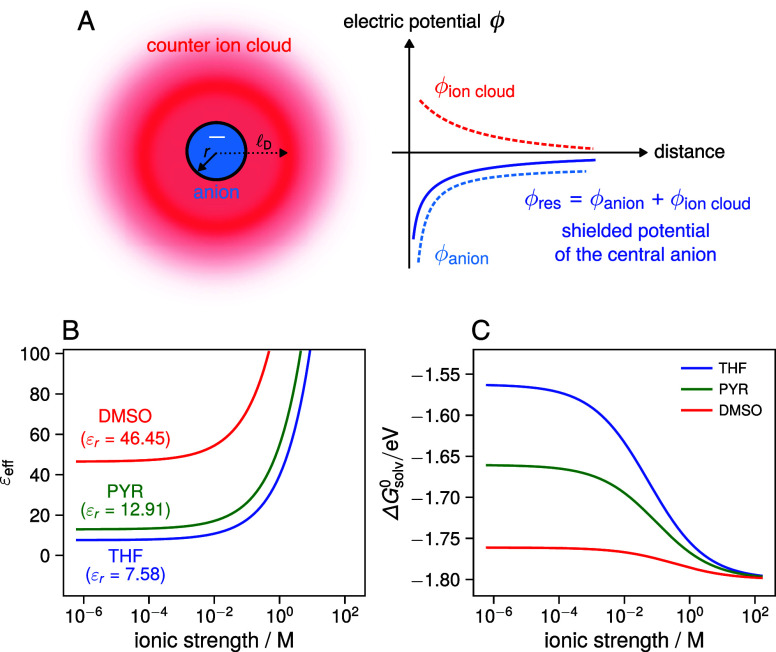
(A) Schematic illustration of the effect of inert counterions on the electrostatic potential of a spherical anion. Counter cations accumulate around the central anion, screening its electrostatic potential and making it less negative. As a result, the electrostatic work required to charge the ion is reduced compared to the case of zero ionic strength, leading to a larger (more stabilizing) solvation energy than predicted by the conventional Born equation. (B) Increase of the effective dielectric constant of the medium with increasing ionic strength and (C) corresponding change in ionic solvation energy, respectively, for three solvents (THF: tetrahydrofuran, PYR: pyridine, DMSO: dimethyl sulfoxide), calculated using the Debye–Hückel–modified Born equation (cf. eq 5) with *r* = 4 Å.

The electrostatic potential of the screened ion can be calculated using Debye–Hückel theory.^41–43^ As detailed in the Supporting Information (SI), evaluating the electrostatic work terms in eq 3 leads to a modified Born expression in which the dielectric constant ε_r_ is replaced by an effective dielectric constant that depends on the ionic strength *I* of the solution:

$$\varepsilon_{\mathrm{eff}}\left(I\right)=\varepsilon_{r}\cdot \left[1+r/l_{D}\right]=\varepsilon_{r}\cdot \left[1+r\cdot \sqrt{\frac{2e^{2}N_{A}I}{\varepsilon_{r}\varepsilon_{0}k_{B}T}}\right]$$ 5

Here, $l_{D}$ is the Debye length, which can be interpreted as the characteristic distance from the center of the ion to the region of maximum counterion concentration surrounding it.

Figure 1B,C show how the effective dielectric constant and Born solvation energy changes according to this modified equation for some common organic solvents of different dielectric constants. For already highly polar solvents such as DMSO (ε_r_ = 46.45 ^25^), the additional increase in the dielectric constant with ionic strength does not alter the solvation energy significantly due to the 1/ε_eff_ proportionality in the Born equation. However, with medium polar solvents like tetrahydrofuran (THF, ε_r_ = 7.58 ^25^) or PYR (ε_r_ = 12.91 ^25^) that exhibit similar dielectric constants as ILs, the solvation energy significantly increases with ionic strength and nearly reaches that of highly polar solvents at molar ionic strengths.

This observation is consistent with a study by Vullev and co-workers,^10^ who showed that reduction potentials in moderately polar solvents such as DCM or THF depend strongly on the concentration of the supporting electrolyte used in electrochemical measurements, whereas no such dependence was observed in highly polar solvents like ACN. They consequently concluded that using reduction potentials measured in DCM or THF with supporting electrolyte in the Weller equation leads to a significant overestimation of the driving force for photoinduced electron transfer in the corresponding pure solvents.

In this study, we aim to experimentally compare solvation energies in prototypical imidazolium-based ILs with those of conventional dipolar organic solvents by analyzing reduction potentials of small organic solutes relevant to photochemistry, measured using cyclic voltammetry (CV). Our results indicate that solvation energies in ILs are indeed comparable to those in polar solvents, likely as a consequence of their high ionic strength. As a result, applying a Born correction in the Weller equation using only the dielectric constant of the IL significantly underestimates the driving force for photoinduced electron transfer reactions in these liquids.

## 2 Principle of the Experiment

Considering the electrochemical reduction of a neutral solute molecule M at an electrode in a given solvent:

$$M_{\left(\mathrm{solvent}\right)}+e_{\left(\mathrm{electrode}\right)}^{-}⇌M_{\left(\mathrm{solvent}\right)}^{-}$$

the experimentally accessible standard reduction potential (*E*_M/M^–^_^0^) can be expressed as^44–52^

$$E_{M/M^{-}}^{0}=F^{-1}\cdot \left[\mathrm{EA}-\left(\Delta G_{\mathrm{solv},M^{-}}^{0}-\Delta G_{\mathrm{solv},M}^{0}\right)\right]+\mathrm{const.}$$ 6

as is also shown in detail in Section 1 of the Supporting Information (SI). Here, *F* is Faraday’s constant, EA the gas-phase electron affinity of M and Δ*G*_solv,i_^0^, the solvation energy of species i. Hence, the value of *E*_M/M^–^_^0^, which can be approximated by the half-wave potential *E*_1/2_ measured in cyclic voltammetry (CV) for an electrochemically reversible system under the common assumption of similar diffusion and activity coefficients of the oxidized and reduced species,^53,54^ directly reflects the difference in solvation energies of the involved species in a given solvent.^10,55^ The constant term in eq 6 corresponds to the absolute potential of the reference electrode used to measure the redox potential.^44–46^ Similarly, considering the electrochemical oxidation of M:

$$M_{\left(\mathrm{solvent}\right)}^{+}+e_{\left(\mathrm{electrode}\right)}^{-}⇌M_{\left(\mathrm{solvent}\right)}$$

it can be shown^10,55^ (cf. SI section 1) that the standard potential *E*_M^+^/M_^0^ can be related to the gas-phase ionization potential (IP) of M as well as to the solvation energies of M^+^ and M via:

$$E_{M^{+}/M}^{0}=F^{-1}\cdot [\mathrm{IP}+(\Delta G_{\mathrm{solv},M^{+}}^{0}-\Delta G_{\mathrm{solv},M}^{0})]+\mathrm{const}.$$ 7

The solvation energies may be crudely estimated using the Born equation (eq 3) which predicts zero solvation energy for neutral species, implying that *E*_M/M^–^_^0^ and *E*_M^+^/M_^0^ shift in direct proportion to the solvation energies of the corresponding ionic species M^–^ and M^+^. In particular, for the reduction, *E*_M/M^–^_^0^ shifts to less negative values (cf. eq 6) as the solvent polarity (ε_r_) increases. This reflects that the reduction of M becomes thermodynamically easier, as M^–^ is stabilized by the larger solvation energy in more polar solvents. Analogously, *E*_M^+^/M_^0^ shifts to less positive values (cf. eq 7) with increasing solvent polarity, indicating that oxidation of M likewise becomes thermodynamically more favorable due to enhanced solvation of M^+^.

The difficulty lies in the experimental measurement and comparison of redox potentials in different solvents. In particular, for a reliable comparison, one must ensure that the constant in eqs 6 and 7, i.e., the absolute potential of the reference electrode, remains unchanged across all solvents. While this condition generally holds true for classical aqueous reference electrodes such as the Ag/AgCl or saturated calomel electrode (SCE), complications arise when using these electrodes in organic solvents.^56^ The presence of a fritted junction between the aqueous reference compartment and the nonaqueous electrolyte introduces an ill-defined liquid junction potential^53,56^ that depends on the solvent. This means that the constant in eqs 6 and 7 includes the liquid junction potential, which varies arbitrarily between different solvents, therefore rendering a direct comparison of redox potentials across solvents impossible. Moreover, aqueous reference electrodes may leak water into the nonaqueous medium, potentially altering or interfering with the electrochemistry of the molecule under investigation.

Alternatively, nonaqueous reference electrodes^56^ based on the Ag/AgNO_3_ couple can be used to minimize or eliminate shifts caused by liquid junction potentials. However, the potential of these electrodes is known to drift, especially in volatile organic solvents, as solvent evaporation alters the Ag^+^ concentration that determines the electrode potential. Instead, so-called pseudoreference electrodes,^56–58^ e.g., a Ag or Pt wire immersed directly in the electrolyte solution, are commonly employed in nonaqueous systems. When using a pseudoreference electrode, one assumes that their potential, which is believed to be determined by a set of poorly defined surface redox equilibria (e.g., Ag/Ag_2_O), does not drift during two quick consecutive CV scans. In practice, one first records the CV of the compound of interest M, for example its reduction (*E*_M/M^–^_^0^). In a subsequent scan, a reference compound with a “well-known” redox potential (typically ferrocene,^57,59^ Fc) is added, and its redox potential *E*_Fc^+^/Fc_^0^ measured. The redox potential of the M/M^–^ couple is then reported relative to Fc^+^/Fc by subtracting *E*_Fc^+^/Fc_^0^ from *E*_M/M^–^_^0^, under the assumption that the reference electrode potential remains stable between the two measurements, i.e., that the constant in eqs 6/7 does not change.

The direct comparison of redox potentials measured in different solvents and reported versus Fc^+^/Fc, however, still remains challenging, as the oxidation potential of ferrocene itself shifts due to differences in the solvation energies of Fc and Fc^+^ across solvents (cf. eq 7). This behavior has been demonstrated experimentally by Noviandri et al.^55^ as well as by Vullev and co-workers,^10^ who showed that *E*_Fc^+^/Fc_^0^ strongly depends on the solvent environment. Nevertheless, subtracting eq 7 from eq 6 illustrates how the reduction potential of the M/M^–^ couple, when referenced to Fc^+^/Fc, depends on the intrinsic molecular properties of the involved chemical species:

$$E_{M/M^{-}}^{0}=F^{-1}\cdot [{\mathrm{EA}}_{M}-{\mathrm{IP}}_{\mathrm{Fc}}-\Delta \Delta G_{\mathrm{solv},M^{-}/M}^{0}-\Delta \Delta G_{\mathrm{solv},{\mathrm{Fc}}^{+}/\mathrm{Fc}}^{0}]$$ 8

Here we have defined ΔΔ*G*_solv,i,j_^0^ as the difference in solvation energies of species *i* and *j*. With this relation at hand, we can now attempt an order-of-magnitude estimate of how the reduction potential *E*_M/M^–^_^0^ vs Fc^+^/Fc shifts as solvent polarity is changed. In particular, if we consider a molecule M similar in size to Fc (assuming a radius of say *r* ≈ 4 Å for all species), all solvation energy terms in eq 8 evaluate to $+\frac{e^{2}}{4\pi \varepsilon_{0}r}\left(1-\frac{1}{\varepsilon_{r}}\right)$ when using the simple Born equation. The left plot in Figure 2 shows how the cyclic voltammogram for the reduction of M, and thus *E*_M/M^–^_^0^, is expected to shift in solvents of different dielectric constants, using IP_Fc_ ≈ 7 eV for ferrocene^60,61^ and EA_M_ ≈ 3 eV for the electron affinity, a typical value for small molecule electron acceptors.^62^

**Figure 2:**
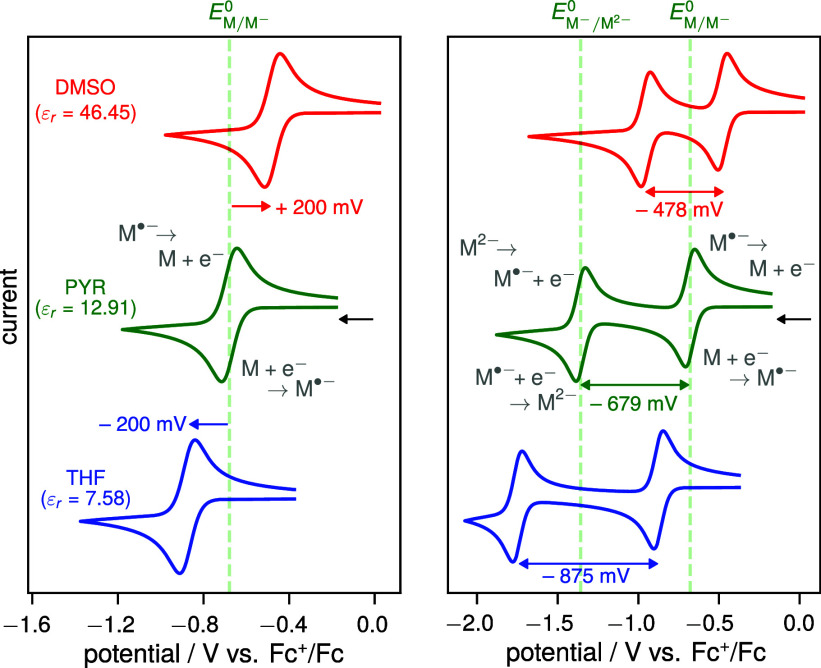
Expected shifts in the cyclic voltammograms for a reversible single (left) and double (right) reduction of a neutral solute M in solvents of different polarity (THF: tetrahydrofuran, PYR: pyridine, DMSO: dimethyl sulfoxide), internally referenced to the Fc^+^/Fc couple. The standard redox potentials were estimated from solvation energies calculated using the Born equation, assuming comparable solvation energies for Fc^+^ and M^–^ with *r* = 4Å for all species, EA_1_ = 3 eV, EA_2_ = −1 eV, and IP_Fc_ = 7 eV. The dashed green lines correspond to the values of *E*_M/M^–^_^0^ and *E*_M^–^/M^2–^_^0^ in PYR to aid comparison.

Pyridine (PYR) was chosen as a reference, as its dielectric constant (ε_r_ = 12.91 ^25^) is similar to that of typical ionic liquids. As expected, the CV in the less polar tetrahydrofuran (THF, ε_r_ = 7.58 ^25^) shifts cathodically, indicating that reduction becomes harder due to a lower solvation energy of M^–^. In contrast, the CV in DMSO shifts anodically, making reduction easier as more solvation energy is gained. In this simple model, shifts on the order of several hundred mV are predicted, which should be readily measurable by CV. Therefore, comparing half-wave potentials measured in conventional solvents versus ILs relative to Fc^+^/Fc should provide a direct experimental handle for solvation energies in ILs.

One potential problem with the approach outlined above is that the solvation energies of Fc^+^ /Fc may differ significantly from those of M and M^–^. In particular, as shown by Noviandri et al.,^55^ the solvation energy difference of Fc^+^ /Fc can depend on the nature of the solvent due to specific solvent–solute interactions such as coordination or π-stacking. However, these differences may be minimized by using a molecule similar in size to Fc and employing noncoordinating solvents. However, this still raises the question of whether there is an experimental handle that is completely independent of the solvation energies of Fc^+^/Fc.

The answer emerges when considering a molecule that can undergo a second reversible reduction:

$$M_{\left(\mathrm{solvent}\right)}^{-}+e_{\left(\mathrm{electrode}\right)}^{-}⇌M_{\left(\mathrm{solvent}\right)}^{2-}$$

characterized by the reduction potential $E_{M^{-}/M^{2-}}^{0}$ . By applying eq 8 twice, it becomes evident that the peak potential separation (Δ*E* ^0^ = *E*_M^–^/M^2–^_^0^ – *E*_M/M^–^_^0^):

$$\begin{array}{ll} \Delta E^{0} & =E_{M^{-}/M^{2-}}^{0}-E_{M/M^{-}}^{0}\\ & =F^{-1}\cdot [{\mathrm{EA}}_{2}-{\mathrm{EA}}_{1}-\Delta \Delta G_{\mathrm{solv},M^{2-}/M^{-}}^{0}+\Delta \Delta G_{\mathrm{solv},M^{-}/M}^{0}] \end{array}$$ 9

between the two reduction waves directly reports on the solvation energies of M and its mono- and dianions, independent of the solvation energies of Fc^+^/Fc. Here, EA_2_ is the second electron affinity of M. Therefore, measuring Δ*E* ^0^ should provide information on the solvation energy, thereby being entirely independent of the internal standard or reference electrode (eq 9 can similarly be obtained by using eq 6 instead of eq 8) used to measure the reduction potentials.

The right plot of Figure 2 illustrates how the peak separation between the two reduction waves changes for the three solvents discussed above, again assuming *r* = 4Å for all species and the Born equation to hold true. The second electron affinity was taken as EA_2_ = – 1 eV as it is typically endothermic to add a second electron to M (negative EA). The plot further shows the absolute positions of the two waves versus the Fc^+^/Fc couple, based on the previous assumption of comparable solvation energies of Fc^+^ and M^–^.

As expected from eq 9, the peak potential separation decreases as the solvent dielectric constant increases (with the above assumptions, all solvation terms in eq 9 reduce to $+\frac{e^{2}}{4\pi \varepsilon_{0}r}\left(1-\frac{1}{\varepsilon_{r}}\right)$ ). Note in particular that the Born equation predicts that the solvation energy of the dianion (M^2–^) is four times that of the monoanion (M^–^). This causes the second reduction wave to shift more strongly than the first as solvent polarity changes when plotted on the Fc^+^/Fc scale. In some extreme cases, such as for the reduction of the carotenoid canthaxanthin,^63^ the dianion solvation energy can be so large that the second reduction becomes thermodynamically easier than the first, leading to a peak potential inversion (positive Δ*E* ^0^) and hence a single CV wave.^53,54,64^ Overall, by studying systems with two reversible reductions, one should have a robust handle to probe and compare solvation energies across different media.

## 3 Methods

### 3.1 Substances

1,2,4,5-tetracyanobenzene (TCB, 97%), tetracyanoquinodimethane (TCNQ, 97%) and ferrocene (Fc, 98%) were purchased from *Sigma-Aldrich*. TCB and Fc were recrystallized from ethanol, and TCNQ was recrystallized from acetonitrile,^65^ prior to use. Electrochemical grade tetrabutylammonium hexafluorophosphate ([nBu_4_N]­[PF_6_], > 98%) was purchased from *Apollo Scientific* and dried in the oven overnight before use.

In order to increase the solubility of the commercially available methyl viologen dichloride (MVCl_2_) in organic solvents, an anion exchange reaction using ammoniumhexafluorophosphate (NH_4_Cl) to yield the corresponding hexafluorophosphate salt was carried out^66^:

$${\mathrm{MVCl}}_{2(\mathrm{aq})}+2{\mathrm{NH}}_{4}{\mathrm{PF}}_{6(\mathrm{aq})}\to \mathrm{MV}({\mathrm{PF}}_{6}{)}_{2(s)}↓+2{\mathrm{NH}}_{4}{\mathrm{Cl}}_{\left(\mathrm{aq}\right)}$$

For this, 460 mg of methyl viologen dichloride hydrate (*Sigma-Aldrich*, 98%) were dissolved in deionized water and a solution containing 730 mg of ammoniumhexafluorophosphate (2.5 eqv., *Sigma-Aldrich*, 98%) in deionized water was added dropwise into the MVCl_2_ solution. The cloudy white precipitate was filtered off and washed several times with deionized water. Recrystallization from a mixture of methanol and acetonitrile (90:10) resulted in faint yellow needles that were dried at 80 °C overnight prior to its use in the CV experiments.

All organic solvents used were of highest commercial purity, and where possible, spectroscopic grade dry solvents were employed. The purity of the solvents for electrochemical measurements were checked by an initial CV scan of the pure solvent/supporting electrolyte solution that yielded only a small capacitive background current for all solvents.

The ionic liquids, 1-ethyl-3-methylimidazolium bis­(trifluoromethylsulfonyl)­imide ([EtMeIm]^+^[Tf_2_N]^−^, >99.5%), 1-ethyl-3-methylimidazolium triflate ([EtMeIm]^+^[TfO]^−^, >97%), 1-ethyl-3-methylimidazolium tetrafluoroborate ([EtMeIm]^+^[BF_4_]^−^, > 99%), 1-ethyl-3-methylimidazolium *n*-octylsulfate ([EtMeIm]^+^[*n*oct-SO_4_]^−^, >98%), 1-ethyl-3-methylimidazolium thiocyanate ([EtMeIm]^+^[SCN]^−^, >98%) and 1-butyl-3-methylimidazolium dicyanamide ([BuMeIm]^+^[N­(CN)_2_]^−^, >98%) were purchased from *IoLiTec* (Germany) and used without further purification. 1-ethyl-3-methylimidazolium dicyanamide ([EtMeIm]^+^[N­(CN)_2_]^−^, >98%) was purchased from *BLDpharm* and used without further purification. The purity of the used ILs was checked in the same way as for the organic solvents by running an initial CV scan in the pure ILs which yielded only a small capacitive background current.

### 3.2 Cyclic Voltammetry

Cyclic voltammograms were measured using a *PalmSens PalmSens4* potentiostat. A three-electrode cell arrangement employing a macroscopic glassy-carbon disk working electrode (*d* = 3 mm) and a platinum wire counter electrode was used. A silver wire was used as a pseudoreference electrode in order to avoid water leakage from aqueous reference electrodes or solvent evaporation and therefore potential drifts common with nonaqueous reference electrodes.^56–58^ All measurements were carried out using a concentration of <5 mM of the molecule under study in the respective organic solvent containing tetrabutylammonium hexafluorophosphate ([nBu_4_N]­[PF_6_], 100 mM) supporting electrolyte or in the pure ionic liquid.^67^ Before addition of the sample, the CV of the pure solvent/supporting electrolyte solution or ionic liquid was measured to ensure that only a small capacitive current was present in the background over the potential range of interest. The solution was purged for several minutes with nitrogen until no oxygen reduction peak was observed in the CV of the neat solvent/supporting electrolyte solution or the pure ionic liquid.

Additionally, the impedance of the cell was measured at high frequency in order to estimate the uncompensated resistance^53,57^ (*R*_u_) of the cell both prior and after addition of the analyte. The obtained value was used as a parameter for electronic compensation for *R*_u_ in the potentiostat software and was augmented until no current oscillations were observed in the CVs.^53^ All CVs were measured at a scan rate of 100 mV/s. For each sample, Fc was added to the solution promptly after the measurement of the CV of the analyte, and its oxidation wave (Fc^+^/Fc) was measured for internal referencing, taking care not to move the experimental arrangement of the electrodes following its addition. A typical measurement series (background, CV of the analyte, CV of Fc) was completed within less than 5 min, minimizing potential drifts of the pseudoreference electrode potential between the analyte and Fc measurements. The working electrode surface was polished using a water alumina (0.3 μm particle size) slurry before and in between measurement sets.

To ensure that the potential of the pseudoreference electrode remained stable over the measurement time, and that no additional overpotentials arise from mass-transport limitations in the viscous ILs or from the choice of electrode material, we performed a series of control experiments also employing a real aqueous fritted Ag/AgCl reference electrode. As detailed in Section 5 of the SI, these measurements demonstrate that the reported half-wave potentials are statistically robust, with a maximum uncertainty of at most 20 mV.

Since in this study, the potential positions of the CV waves were of interest rather than the absolute magnitude of the current (controlled by the concentration/diffusion coefficient^54^ of the analyte in the respective solvent), CVs were normalized with respect to their first cathodic peak in order to aid comparison among the different solvents/ILs used.

### 3.3 Quantum Chemical Calculations

All density functional theory quantum chemical calculations were performed using Gaussian 16 ^68^ employing the range-separated hybrid functional CAM-B3LYP in combination with the aug-cc-pVDZ basis set. For a detailed discussion of how reduction potentials vs Fc^+^/Fc are computed see SI section 3.

## 4 Results and Discussion

We now turn to the experimental implementation of the ideas outlined above. For this, we studied the electrochemical reductions of three small organic molecules, namely, 1,2,4,5-tetracyanobenzene (TCB), tetracyanoquinodimethane (TCNQ), and methyl viologen (MV^2+^). These compounds exhibit either one (TCB) or two consecutive (TCNQ, MV^2+^) electrochemically reversible reductions which allows us to compare their half-wave potentials measured in different media and thus to gain insight into the solvation energies in ILs compared to conventional solvents.

### 4.1 Tetracyanobenzene—TCB

The CVs of the electrochemical reduction of TCB measured in the three organic solvents discussed above as well in the imidazolium based ionic liquid 1-ethyl-3-methylimidazolium dicyanamide ([EtMeIm]^+^[N­(CN)_2_]^−^, ε_r_ = 11.0 ^24^) are shown in Figure 3.

**Figure 3:**
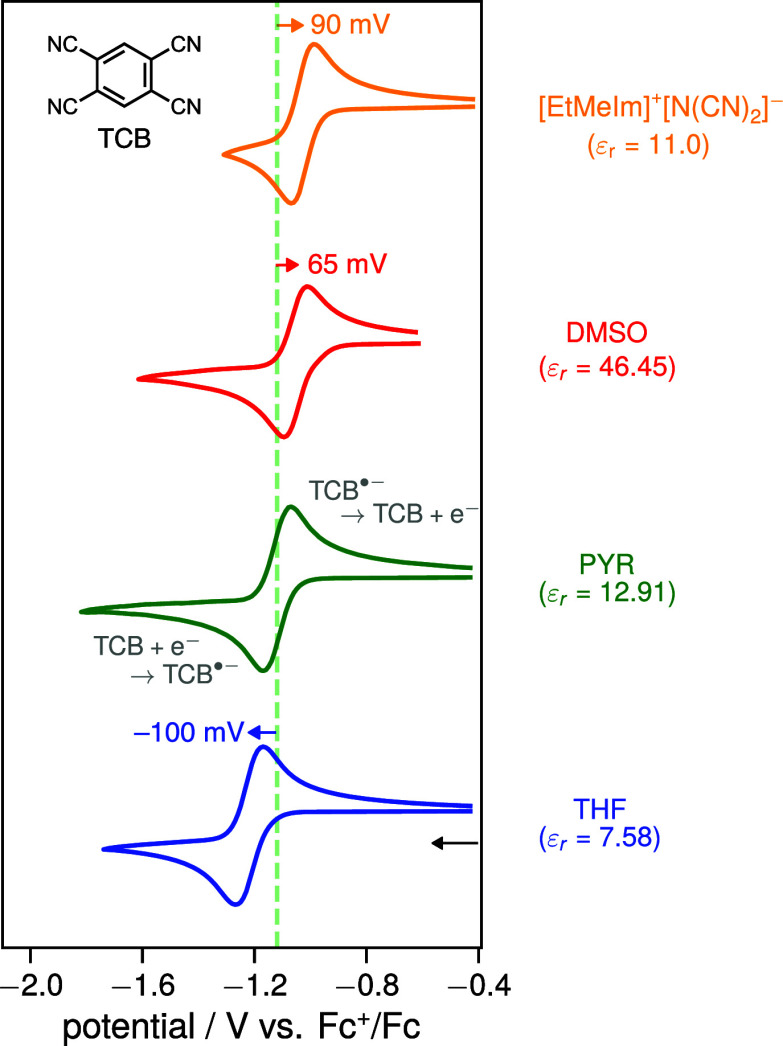
Normalized cyclic voltammograms of the reduction of TCB in three organic solvents (THF: tetrahydrofuran, PYR: pyridine, DMSO: dimethyl sulfoxide) and the imidazolium-based ionic liquid [EtMeIm]^+^[N­(CN)_2_]^−^ recorded at a glassy carbon working electrode internally referenced vs Fc^+^/Fc at a scan rate of 100 mV/s. For the organic solvents, 100 mM of [nBu_4_N]^+^[PF_6_]^−^ was added as supporting electrolyte. For further experimental details as well as solvent parameters and chemical structures, see sections 2 and 3 of the SI. The dashed green line corresponds to the half-wave potential *E*_1/2_ ≈ *E*_TCB/TCB^•–^_^0^ in PYR to aid comparison.

In all media, a single reversible CV wave is observed, which shows the reduction of TCB to its radical anion TCB^•–^ on the forward scan and its reoxidation on the reverse scan:

$$\begin{array}{ll} \mathrm{TCB}+e^{-}⇌{\mathrm{TCB}}^{•-} & E_{1/2}\approx E_{\mathrm{TCB}/{\mathrm{TCB}}^{•-}}^{0} \end{array}$$

As expected (cf. Figure 2, left), the reduction wave in DMSO is shifted anodically (by approximately 65 mV), while that in THF is shifted cathodically (by about −100 mV) relative to the CV in PYR. These shifts reflect the larger and smaller gains in solvation energy of TCB^•–^ in DMSO and THF with respect to that in PYR respectively.

Most striking, however, is the anodic shift of approximately 90 mV of the reduction wave measured in the ionic liquid, which even exceeds the shift observed in DMSO, indicating a comparable or possibly even larger solvation energy in the ionic liquid than in DMSO. According to the dielectric constant of [EtMeIm]^+^[N­(CN)_2_]^−^, the wave should appear at roughly the same position as, or even slightly cathodically shifted relative to, that observed in PYR. The experimental results therefore suggest that the solvation energy in the ionic liquid is significantly larger than that in PYR, despite their similar dielectric constants.

To verify whether these observed trends are general, we measured the CVs of TCB in eight dipolar organic solvents and six imidazolium-based ILs (for solvent abbreviations, structures, and dielectric constants, see Table S1, SI). The resulting voltammograms are shown in Figure 4. In all investigated ILs, the TCB reduction wave is observed at significantly more positive potentials compared to that in PYR. The half-wave potentials in the ILs are generally similar to, or in some cases even more positive than, those measured in DMSO, indicating that the solvation energies in these ILs are comparable those of highly polar solvents such as DMSO.

**Figure 4:**
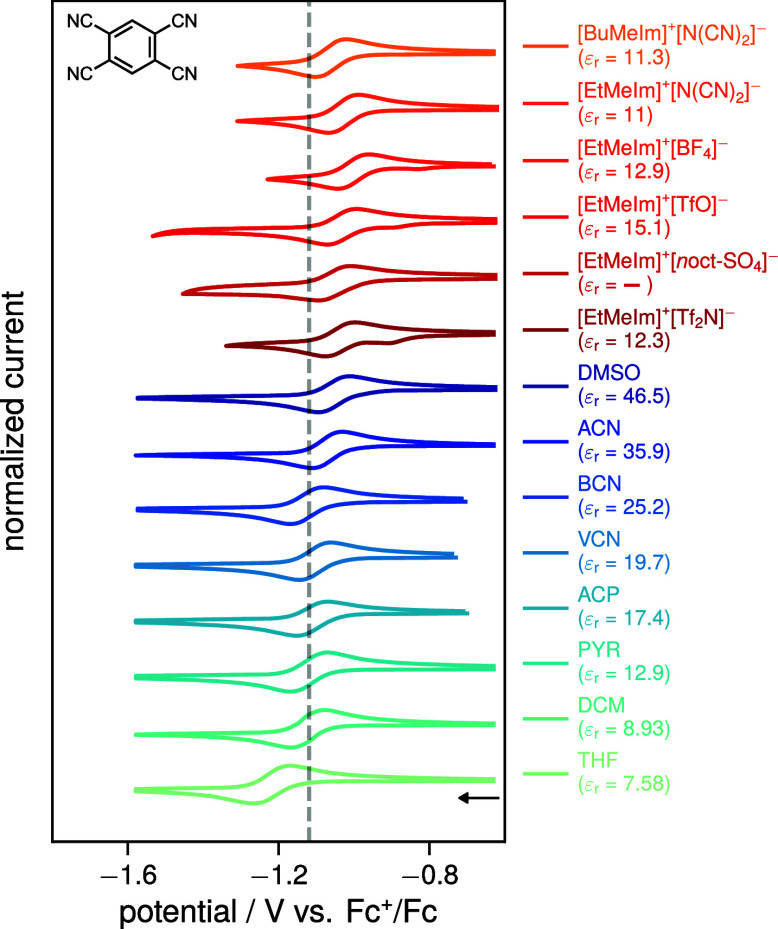
Normalized cyclic voltammograms of the reduction of TCB in various organic solvents (blue shades) and ionic liquids (red shades) recorded at a glassy carbon working electrode internally referenced vs Fc^+^/Fc at a scan rate of 100 mV/s. For the organic solvents, 100 mM of [nBu_4_N]^+^[PF_6_]^−^ was added as a supporting electrolyte. For further experimental details as well as solvent structures/abbreviations and dielectric constants, see section 2 and 3 of the SI. The dashed gray line corresponds to the half-wave potential *E*_1/2_ ≈ *E*_TCB/TCB^•–^_^0^ in PYR to aid comparison.

The largest anodic shift is observed for the ionic liquid [EtMeIm]^+^[BF_4_]^−^ (cf. Table S1), which is composed of the smallest ions of the investigated ILs. As shown in a recent molecular dynamics study,^39^ specific solvation in ILs is linked to charge oscillation and is predicted to be most pronounced in systems composed of “pure” monopoles, such as in the case of molten NaCl. In typical ILs, these charge oscillations are partially damped due to screening by the permanent dipoles of their molecular ions. Consequently, ILs with smaller ions are expected to exhibit weaker dipolar screening, leading to stronger solvation for solutes that fit within the “gaps” of the charge oscillations, which are computed to be most relevant for solutes with sizes of 2–4 Å, consistent with the size of TCB. This explanation would also explains why, for the larger [BuMeIm]^+^[N­(CN)_2_]^−^, the reduction wave is shifted to more negative potentials compared to that in [EtMeIm]^+^[N­(CN)_2_]^−^, indicating a smaller solvation energy.

According to the Born model (cf. eq 3), the solvation terms in eq 8 should be inversely proportional to the dielectric constant and hence the position of the half-wave potential should also vary proportionally to the inverse of ε_r_. Figure 5 shows the correlation between the measured half-wave potential *E*_1/2_ for the reduction of TCB against 1/ε_r_ for the various organic solvents and ILs. While, the conventional dipolar organic solvents exhibit an approximately linear trend with 1/ε_r_, with larger deviations as the solvent polarity decreases (see below), the ILs depart strongly from this trend, indicating a substantial divergence from the standard Born model. To reconcile their behavior with that of the regular organic solvents, one would have to assume a much larger “effective” dielectric constant than the bulk macroscopic values reported for these liquids as expected from the discussion in Figure 1.

**Figure 5:**
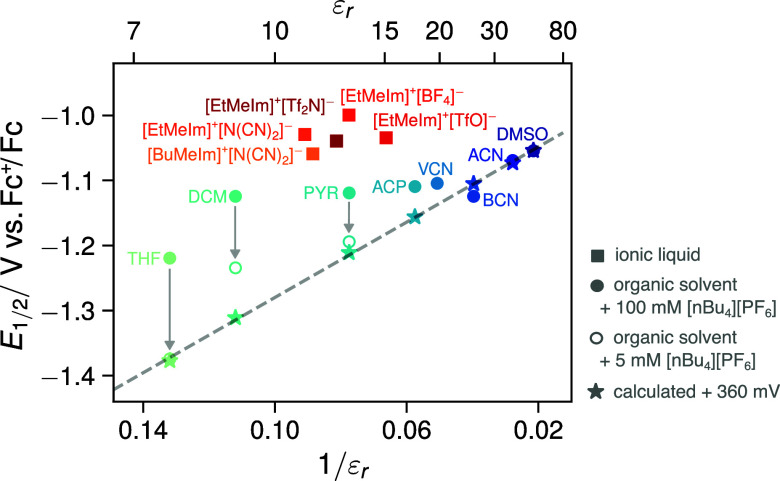
Halfwave potentials (*E*_1/2_ ≈ *E*_TCB/TCB^•–^_^0^) of the reduction of TCB in various organic solvents (blue shaded circles) and ionic liquids (red shaded squares) vs the inverse of their respective dielectric constant (1/ε_r_). The stars represent values obtained from DFT calculations (CAM-B3LYP/aug-cc-pVDZ, for details, see SI sections 3 and 4) shifted by +360 mV, employing a polarizable continuum model to calculate solvation energies in the organic solvents.

We performed several control experiments (see SI Section 5) to ensure that the observed half-wave potential shifts are statistically robust. Measurements were repeated using different electrode materials and a real fritted aqueous Ag/AgCl reference electrode instead of a silver wire pseudo reference electrode. When internally referenced to Fc^+^/Fc, the half-wave potentials in the various solvents and ILs quantitatively agree with the halfwave potentials shown Figure 4. Additionally, scan rate dependence studies in the ILs confirmed that the observed potentials are diffusion-controlled, with no additional overpotential from mass-transport limitations in these viscous media and an independence of *E*_1/2_ on the scan rate.

We further tried to rationalize the observed trend for the organic solvents with the help of some basic density functional theory (DFT) calculations (see SI, section 3). In these calculations, all terms in eq 8 were evaluated quantum chemically and a polarizable continuum model (PCM) was used for the solvation energies. This approach should be more sophisticated than the spherical approximation of the Born model while still modeling the solvent as a dielectric continuum. To align the computed redox potentials with the experimental potential measured in DMSO, an absolute shift of +360 mV needed to be applied to the calculated values (cf. Table S7, SI) which likely arises from uncertainties in the calculation of electron affinities and ionization potentials. After this correction, the agreement between the calculated (stars in Figure 5) and experimental values for the most polar solvents, ACN and benzonitrile (BCN), is excellent. As expected from a continuum solvation model, like the Born model, the calculated values follow a linear relationship with 1/ε_r_.

While the experimental data fits well to this trend for highly polar solvents, stronger deviations are observed as the solvent polarity decreases. These deviations can be attributed to the larger increase of solvation energy and larger effective dielectric constant with increasing ionic strength due to the supporting electrolyte in the low polarity solvents (cf. Figure 1 C). Indeed, Vullev and co-workers^10^ report that the “effective” dielectric constant of dichloromethane (DCM) increases from 8.93 to 14.3 in the presence of 100 mM [nBu_4_N]^+^ [PF_6_]^−^. To verify this hypothesis, we measured the half-wave potentials in the three least polar solvents, THF, DCM and PYR, using a minimal supporting electrolyte concentration of 5 mM [nBu_4_N]^+^[PF_6_]^−^ (Figure S1, SI). As expected, the recorded CV waves shift cathodically to more negative potentials as the electrolyte concentration is reduced. The relatively large shifts of up to nearly −200 mV highlight the strong influence of the ionic strength in these low-polarity solvents, i.e., showing how the effective polarity and solvation energy increase in the presence of a supposedly inert electrolyte salt. Moreover, when using the half-wave potentials measured in the 5 mM [nBu_4_N]^+^[PF_6_]^−^ solutions, the previously strongly deviating data points approach the predicted theoretical linear trend for the organic solvents (cf. Figure 5, open circles). The points for THF and PYR are particularly remarkable, as they almost perfectly align with the calculated trend.

### 4.2 Tetracyanoquinodimethane—TCNQ

To assess whether these trends are general, we now turn to a molecule that undergoes two reversible reductions, namely TCNQ. Figure 6 shows the cyclic voltammograms of the reduction of TCNQ in the three organic solvents THF, PYR, and DMSO, as well as in the ionic liquid [EtMeIm]^+^[N­(CN)_2_]^−^. In all media, two reversible waves are observed, corresponding to the first,

$$\begin{array}{ll} \mathrm{TCNQ}+e^{-}⇌{\mathrm{TCNQ}}^{•-} & E_{1/2}^{1}\approx E_{\mathrm{TCNQ}/{\mathrm{TCNQ}}^{•-}}^{0} \end{array}$$

and second,

$$\begin{array}{ll} {\mathrm{TCNQ}}^{•-}+e^{-}⇌{\mathrm{TCNQ}}^{2-} & E_{1/2}^{2}\approx E_{{\mathrm{TCNQ}}^{•-}/{\mathrm{TCNQ}}^{2-}}^{0} \end{array}$$

reductions of TCNQ. As described in the introduction, the separation between the first (*E*_1/2_^1^) and second (*E*_1/2_^2^) half-wave potential reflects the difference in solvation energies of TCNQ and its mono- and dianions, while being independent of the solvation energies of the Fc^+^/Fc reference couple (cf. eq 9). Furthermore, a decrease in the magnitude of the half-wave potential separation (Δ*E*_1/2_ = *E*_1/2_^2^ – *E*_1/2_^1^) indicates an increase in solvation energy (cf. Figure 2, right). This is precisely what is observed experimentally, i.e., |Δ*E*_1/2_| increases in THF and decreases in DMSO relative to PYR, in line with their respective lower and higher solvation energies. Notably, the ionic liquid exhibits an even smaller potential separation than that in DMSO, again indicating a solvation energy comparable to or exceeding that of highly polar solvents consistent with the behavior observed for TCB.

**Figure 6:**
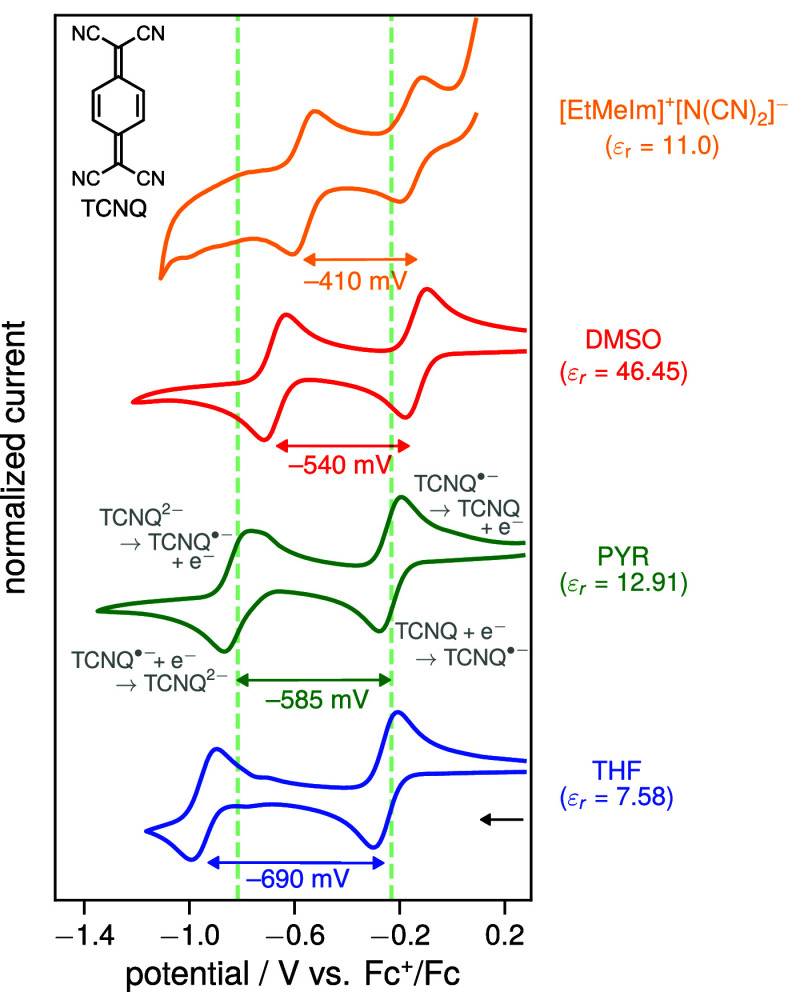
Normalized cyclic voltammograms of the reduction of TCNQ in three organic solvents (THF: tetrahydrofuran, PYR: pyridine, DMSO: dimethyl sulfoxide) and the imidazolium-based ionic liquid [EtMeIm]^+^[N­(CN)_2_]^−^ recorded at a glassy carbon working electrode internally referenced vs Fc^+^/Fc at a scan rate of 100 mV/s. For the organic solvents, 100 mM of [nBu_4_N]^+^[PF_6_]^−^ was added as supporting electrolyte. For further experimental details as well as solvent parameters and chemical structures, see section 2/3 of the SI. The dashed green line corresponds to the half-wave potentials for the first (*E*_1/2_^1^ ≈ *E*_TCNQ/TCNQ^•–^_^0^) and second (*E*_1/2_^2^ ≈ *E*_TCNQ^•–^/TCNQ^2–^_^0^) reduction in PYR to aid comparison.

The CV recorded in [EtMeIm]^+^[N­(CN)_2_]^−^ appears slightly distorted due to the onset of the oxidation of the ionic liquid at the most positive potentials. This current rise is also observed in the CV of the pure IL. Considering the absolute positions of the reduction waves on the Fc^+^/Fc scale, the experimental data follow the predicted trends from eq 8. In particular, assuming similar solvation energies for Fc^+^ and TCNQ^•–^, the first half-wave potential is expected to shift anodically with increasing solvent polarity, as is observed experimentally, similar to the measurements in TCB. Notably, the second half-wave potential, which reflects the solvation energy of the dianion (TCNQ^2–^), exhibits an even larger shift. According to the Born model (cf. eq 3), the solvation energy of the TCNQ^2–^ is expected to be four times as large as that of the TCNQ^•–^. Thus, as clearly seen in the data, the absolute anodic shift of *E*_1/2_^2^ compared to that of *E*_1/2_^1^ is significantly larger, which leads to the reduction of |Δ*E*_1/2_| as polarity increases.

We further recorded the CVs for the reduction of TCNQ in various other organic solvents and ILs, as shown in Figure S2 (SI). In all investigated ILs, the observed half-wave potential separations are either comparable to or smaller than those measured in DMSO, once again indicating that solvation energies in these liquids are similar to those of highly polar solvents. Moreover, the positions of the first and second half-wave potentials, as well as Δ*E*_1/2_, scale approximately linearly with 1/ε_r_ for the organic solvents, while the ILs strongly deviate from this trend (cf. Figure S3, SI). The data obtained in the organic solvents is again in line with theoretical DFT calculations, with the largest deviations from the calculated linear behavior observed for the least polar solvents due to the previously discussed salt effect.

### 4.3 Methyl Viologen—MV^2+^

As a final test of the enhanced solvation energies in ionic liquids compared to conventional organic solvents of similar dielectric constants, we examined a third molecule that also undergoes two reversible reductions, namely methyl viologen (MV^2+^). Unlike TCB and TCNQ, MV^2+^ is initially doubly positively charged, which results in exactly the opposite trend as for TCB/TCNQ, i.e., a shift of the first reduction wave to more negative potentials as solvent polarity increases. This is because upon its first reduction,

$$\begin{array}{ll} {\mathrm{MV}}^{2+}+e^{-}⇌{\mathrm{MV}}^{•+} & E_{1/2}^{1}\approx E_{{\mathrm{MV}}^{2+}/{\mathrm{MV}}^{•+}}^{0} \end{array}$$

the large solvation energy of MV^2+^ (approximately four times that of MV^•+^ according to the Born model) is lost. Consequently, reduction requires more energy in more polar solvents, resulting in a cathodic shift of the CV wave with increasing polarity. This behavior directly follows from eq 8 as shown in detail in the SI section 4.3. Furthermore, eq 8 predicts that the second reduction,

$$\begin{array}{ll} {\mathrm{MV}}^{•+}+e^{-}⇌\mathrm{MV} & E_{1/2}^{2}\approx E_{{\mathrm{MV}}^{•+}/\mathrm{MV}}^{0} \end{array}$$

should be largely independent of solvent polarity if one assumes similar solvation energies for Fc^+^ and MV^•+^. However, if the first wave shifts cathodically while the second remains nearly constant, the separation of the two half-wave potentials |Δ*E*_1/2_| (which, as for TCNQ, is independent of the solvation energies of Fc and Fc^+^) is expected to again decrease with increasing solvent polarity (cf. eq s27, SI).

Figure 7 shows the experimental CVs for the reduction of MV^2+^ in the two organic solvents PYR and DMSO as well as in the ionic liquid [EtMeIm]^+^[N­(CN)_2_]^−^. In all media, two reversible CV waves corresponding to the first and second reduction of MV^2+^ are observed. Unfortunately, due to the limited solubility of MV^2+^ in THF and DCM and/or electrochemically irreversible behavior in these solvents, these low-polarity solvents are not included in the measurement series. Nonetheless, the CVs shown in Figure 7 once again indicate that the solvation energy in [EtMeIm]^+^[N­(CN)_2_]^−^ is comparable to that in DMSO, as evidenced by the similar in magnitude, and smaller half-wave potential separation between the two reduction waves compared to the CV in PYR. Note further, that the absolute shifts of the waves on the Fc^+^/Fc scale follow the expectation outlined above, i.e., while the second reduction wave remains largely unaffected by the medium, the first wave shifts cathodically to more negative potentials in both DMSO and the ionic liquid, highlighting the larger solvation energy of MV^2+^ in these media compared to PYR.

**Figure 7:**
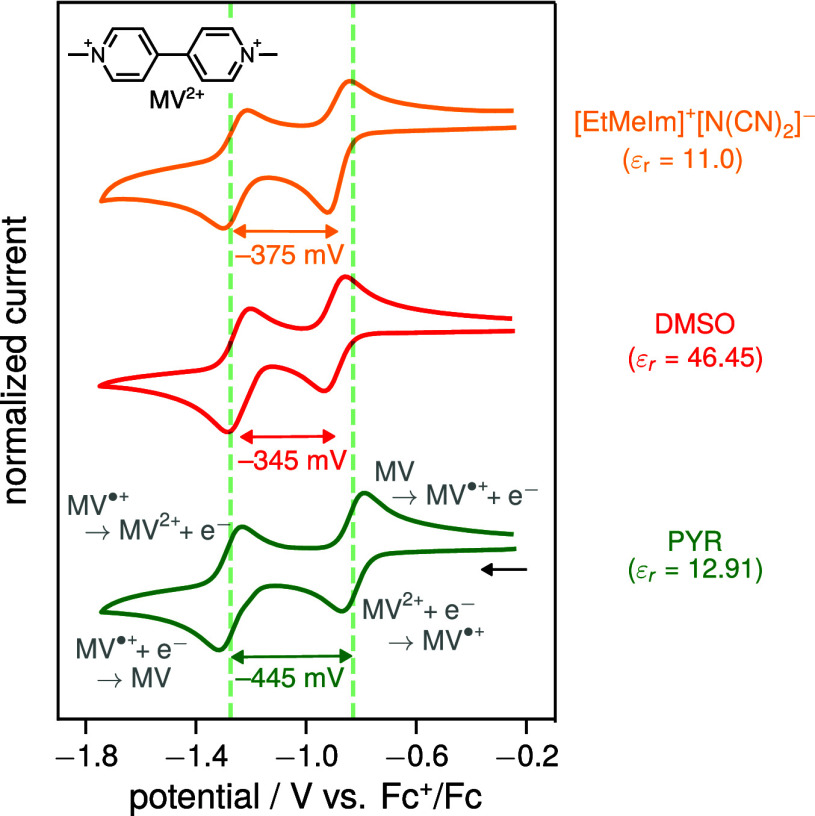
Normalized cyclic voltammograms of the reduction of [MV^2+^]­[(PF_6_^–^)_2_] in the organic solvents pyridine (PYR) and dimethyl sulfoxide (DMSO) and the imidazolium-based ionic liquid [EtMeIm]^+^[N­(CN)_2_]^−^ recorded at a glassy carbon working electrode internally referenced vs Fc^+^/Fc at a scan rate of 100 mV/s. For the organic solvents, 100 mM of [nBu_4_N]^+^[PF_6_]^−^ was added as supporting electrolyte. For further experimental details as well as solvent parameters and chemical structures, see section 3.2 as well as section 2 of the SI. The dashed green line corresponds to the half-wave potentials for the first (*E*_1/2_^1^ ≈ *E*_MV^2+^/MV^•+^_^0^) and second (*E*_1/2_^2^ ≈ *E*_MV^•+^/MV_^0^) reduction in PYR to aid comparison.

We further measured the CVs of MV^2+^ in a total of six organic solvents and five ILs as shown in Figure S4 (SI). In all ILs, the half-wave potential separation is close to that observed in ACN or DMSO and is significantly smaller than that in PYR. Moreover, the first reduction wave is consistently shifted cathodically relative to the measurement in PYR. Quantum-chemical calculations further reproduce these trends (cf. Figure S5, SI), with larger deviations again appearing for the less polar solvents. Overall, these results again indicate that the solvation energy in ILs is comparable to that of polar organic solvents.

## 5 Conclusions

Herein, we presented a comparative study of the solvation energies of several imidazolium-based ILs relative to conventional dipolar organic solvents by analyzing the half-wave reduction potentials, measured via cyclic voltammetry, of three typical small organic solute molecules exhibiting either one or two reversible reductions. We demonstrated that the positions of the half-wave potentials, as well as the half-wave potential separations in systems undergoing two consecutive reductions—which we show to be independent of the solvation energies of the internal standard used to calibrate the potential axis, i.e., Fc^+^/Fc— directly report on the solvation energies of the species involved in the reduction(s) in the given medium. For all three molecules studied, the reduction potentials in conventional organic solvents shift in line with their bulk dielectric constants (ε_r_), following an approximately linear trend with 1/ε_r_, as predicted by continuum solvation models such as the Born equation. Stronger deviations from the expected trend are observed for the least polar solvents, which exhibit higher solvation energies than anticipated. These deviations can be attributed to the presence of supporting electrolyte salts, which lead to electrostatic screening and thus to a pronounced ionic-strength dependence of the solvation energy, an effect that is much less significant in highly polar solvents (cf. Figure 1).

Ionic liquids represent an extreme case, as they are essentially molten salts with molar ionic strengths. Hence, the dependence of solvation energy on ionic strength cannot be neglected. This results in a substantially larger effective dielectric constant that should be used in the Born equation, leading to solvation energies comparable to those of strongly polar solvents such as ACN and DMSO. This interpretation is supported by the experimental data for the three investigated molecules, all of which show half-wave potential shifts comparable to, or even exceeding, those observed in highly polar solvents such as DMSO. These strong deviations from the expected 1/ε_r_ trend indicate significantly enhanced solvation energies and a much higher effective polarity than implied by the dielectric constants of the ILs alone. The macroscopically measurable dielectric constant of ILs may primarily reflect the dipolar character of their constituent ions. Indeed, the dielectric constant of imidazole (ε_r_ = 23.0) is comparable, although larger, than that of imidazolium-based ILs. Accordingly, pure imidazole containing a molar concentration of inert salt would likewise be expected to exhibit solvation energies similar to those of highly polar solvents, consistent with the trend shown in Figure 1C.

Finally, we note that the present analysis considers only nonspecific solute–solvent interactions. Specific interactions such as hydrogen bonding or ion pairing are also likely to contribute to solvation in ILs, and solvation energies in ILs are known to strongly depend on solute size.^39^ Nevertheless, the main conclusion from a photochemical perspective remains unchanged: for typical small organic molecules relevant to photochemistry, charge-separation driving forces in ILs are significantly larger than expected based solely on their dielectric constants. Using the standard Born correction in the Weller equation therefore underestimates the driving force and may lead to misinterpretation of experimental results. More reliable estimates can be obtained by employing a Born correction that uses an effective dielectric constant accounting explicitly for ionic strength (cf. eq 5).

## Supplementary Material



## Data Availability

The data supporting the findings of this study is available on public repository and can be downloaded at: 10.26037/yareta:vmuuwzu7bnhshak2ulr3weqe2a

## Notes

Theoretical background and derivation of eq 5, experimental details, solvent/ionic liquid properties, quantum chemical calculations, additional CV/calculated data, and control experiments (PDF)
