# Supplementary material for: When Dielectric Constants Deceive: Interrogating Solvation in Ionic Liquids with Cyclic Voltammetry
Source: J Phys Chem B. 2026 Feb 26;130(10):2897–907. doi: 10.1021/acs.jpcb.6c00284 (PMC12990102; doi:10.1021/acs.jpcb.6c00284)
Supplement: Supplementary file 1 [file jp6c00284_si_001.pdf]

SUPPORTING INFORMATION FOR:

When Dielectric Constants Deceive: Interrogating Solvation in Ionic Liquids with Cyclic Voltammetry

Johannes Wega,*[a], Franck Guignard[a] and Eric Vauthey[a]

[a] *Departement of Physical Chemistry, Quai-Ansermet 30, 1205 Geneva, Switzerland.*

**Email:* johannes.wega@unige.ch

Contents

Page

1. Theoretical Background S2
   1.1. Born Model S2
   1.2. Effect of Ionic Strength on the Born Model S2
   1.3. Standard Reduction Potentials S3
2. Solvent / Ionic Liquid Properties S4
3. Quantum Chemical Calculations S5
4. Additional Data S7
   4.1. Tetracyanobenzene - TCB S7
   4.2. Tetracyanoquinodimethane - TCNQ S8
   4.3. Methyl viologen - MV^{2+} S9
5. Control Experiments S11
6. References S12

1. Theoretical Background

1.1 Born Model

Ionic solvation energies can, in a first approximation, be estimated by the Born equation (Eq. 3 main text) where the chemical species are considered to be spheres. In his model, Born defines the solvation energy as the difference in energy required to charge up a sphere of radius r with a charge $z \cdot e$ in vacuum ($\varepsilon_r = 1$) versus the same process in a solvent with continuous dielectric constant ε_r, i.e.:[1],

$$\Delta G^0_{\text{solv}} = W(\varepsilon_r) - W(\varepsilon_r = 1) \tag{s1}$$

Any changes in entropy upon solvation are not considered. The energy required to reversibly charge up a sphere with a charge of $q = z \cdot e$ can be calculated using:

$$W = \int_0^{ze} \phi(q)\,\mathrm{d}q \tag{s2}$$

where ϕ is the electrostatic potential of a charged sphere with radius r:[2]

$$\phi = \frac{q}{4\pi\varepsilon_0\varepsilon_r r} \tag{s3}$$

Performing the integration yields:

$$W = \frac{z^2 e^2}{8\pi\varepsilon_0\varepsilon_r r} \tag{s4}$$

Using this relation in Eq. s1 gives the Born equation.

1.2 Effect of Ionic Strength on the Born Model

As explained in the main text, the presence of inert counter-ions leads to the formation of a counter-ion cloud that screens the electrostatic potential of a central spherical ion. According to Debye–Hückel theory,[3,4] the electrostatic potential of the screened sphere at its surface is given by:

$$\phi_{\text{DH}} = \frac{q}{4\pi\varepsilon_{\text{r}}\varepsilon_0 r} \cdot \frac{1}{1 + r/\ell_{\text{D}}} \tag{s5}$$

where ℓ_{D} is the Debye-length:

$$\ell_{\text{D}} = \sqrt{\frac{\varepsilon_{\text{r}}\varepsilon_0 k_{\text{B}} T}{2e^2 N_{\text{A}} I}} \tag{s6}$$

Here, N_{A} is Avogadro's constant, k_{B} the Boltzmann constant, T the temperature, and I the ionic strength of the solution. The electrostatic work required to charge the sphere to $q = ze$ is then:

$$W = \int_0^{ze} \phi_{\text{DH}}(q)\,\mathrm{d}q = \frac{z^2 e^2}{8\pi\varepsilon_{\text{r}}\varepsilon_0\, r\,(1 + r/\ell_{\text{D}})} \tag{s7}$$

Following the same approach as in the Born model, the solvation free energy then becomes:

$$\Delta G^0_{\text{solv}} = W(\varepsilon_r, I) - W(\varepsilon_r = 1) \tag{s8}$$

$$= -\frac{z^2 e^2}{8\pi\varepsilon_0\, r}\left(1 - \frac{1}{\varepsilon_{\text{eff}}(I)}\right) \quad \text{with} \quad \varepsilon_{\text{eff}}(I) = \varepsilon_{\text{r}} \cdot [1 + r/\ell_{\text{D}}(I)] \tag{s9}$$

This expression has the same form as the conventional Born equation, but with the dielectric constant replaced by an effective dielectric constant $\varepsilon_{\text{eff}}(I)$ that increases with ionic strength.

1.3 Standard Reduction Potentials

The goal of this subsection is to illustrate how the standard reduction potentials (E^0) of a solute M relate to its electron affinity (EA) for reduction, its ionization potential (IP) for oxidation, and the solvation energies of the species involved, thereby deriving the equations presented in the main text. The derivations outlined here were adapted from Refs. 5–8.

Let us begin with the electrochemical reduction of a neutral solute M in a solvent at an electrode:

$$\mathrm{M_{(solvent)}} + \mathrm{e^-_{(electrode)}} \rightleftharpoons \mathrm{M^-_{(solvent)}} \qquad E^0_{\mathrm{M/M^-}} \tag{s10}$$

characterized by the standard redox potential $E^0_{\mathrm{M/M^-}}$. From general electrochemistry, the Gibbs free-energy of reaction ΔG^0 of an electrochemical equilibrium, such as that in Eq. s10, is related to the reduction potential via:[9,10]

$$\Delta G^0 = -F E^0 \tag{s11}$$

where F is Faraday's constant. The reduction potential $E^0_{\mathrm{M/M^-}}$ is normally measured experimentally against some reference electrode which has a constant absolute potential. This introduces an off-set in the absolute value of ΔG^0 with respect to vacuum. From standard thermodynamics one can also write the free-energy of reaction s10 as the sum of the free-energies of the products minus the sum of the free-energies of the reactants, i.e.:

$$\Delta G^0 = G^0_{\mathrm{liq,M^-}} - G^0_{\mathrm{liq,M}} + C \tag{s12}$$

where C is a constant due to the aforementioned energy off-set which is specific to the reference electrode. Next, we proceed by writing the free-energy of a species in solution as its free energy in vacuum (vac) plus the energy that is gained when dissolving the species in a liquid (liq), i.e. the *solvation energy*:

$$G^0_{\mathrm{liq,M}} = G^0_{\mathrm{vac,M}} + \Delta G^0_{\mathrm{solv,M}} \tag{s13}$$

$$G^0_{\mathrm{liq,M^-}} = G^0_{\mathrm{vac,M^-}} + \Delta G^0_{\mathrm{solv,M^-}} \tag{s14}$$

With these two relations Eq. s12 becomes:

$$\Delta G^0 = G^0_{\mathrm{vac,M^-}} - G^0_{\mathrm{vac,M}} + (\Delta G^0_{\mathrm{solv,M^-}} - \Delta G^0_{\mathrm{solv,M}}) + C \tag{s15}$$

The first part of this equation is simply the reaction free-energy of adding an electron to M in vacuum and is experimentally accessible from the electron affinity (EA) of M, which is defined as the exothermicity ($-\Delta H^0$) of the reaction:[6]

$$\mathrm{M_{(vac)}} + \mathrm{e^-_{(vac)}} \rightarrow \mathrm{M^-_{(vac)}} \qquad \Delta H^0 = -\mathrm{EA} \tag{s16}$$

Neglecting entropic changes upon reduction of M to $\mathrm{M^-}$, allows one to replace $G^0_{\mathrm{vac,M^-}} - G_{\mathrm{vac}}$ by $-$EA in Eq. s15, yielding:

$$\Delta G^0 = -\mathrm{EA} + (\Delta G^0_{\mathrm{solv,M^-}} - \Delta G^0_{\mathrm{solv,A}}) + C \tag{s17}$$

Inserting this relation into Eq. s11 gives Eq. 6 of the main text with const. $= -F^{-1}C$, thus revealing how $E^0_{\mathrm{M/M^-}}$ is related to the electron affinity and the differences in solvation energies of $\mathrm{M^-}$ and M.

Following the same steps for an electrochemical oxidation, i.e.:

$$\mathrm{M^+_{(solvent)}} + \mathrm{e^-_{(electrode)}} \rightleftharpoons \mathrm{M_{(solvent)}} \qquad E^0_{\mathrm{M^+/M}} \tag{s18}$$

gives:

$$\Delta G^0 = G^0_{\mathrm{vac,M}} - G^0_{\mathrm{vac,M^+}} - (\Delta G^0_{\mathrm{solv,M^+}} - \Delta G^0_{\mathrm{solv,M}}) + C \tag{s19}$$

for the reaction free-energy. Here, the first term can be related to the ionization potential (IP) of M:

$$\mathrm{M_{(vac)}} \rightarrow \mathrm{M^+_{(vac)}} + \mathrm{e^-_{(vac)}} \qquad \Delta H^0 = \mathrm{IP} \tag{s20}$$

Again neglecting any entropic changes gives:

$$\Delta G^0 = -\mathrm{IP} - (\Delta G^0_{\mathrm{solv,M^+}} - \Delta G^0_{\mathrm{solv,M}}) + C \tag{s21}$$

Similarly inserting this into Eq. s11 gives Eq. 2 of the main text which shows how $E^0_{\mathrm{M^+/M}}$ is related to the ionization potential and the solvation energy difference of $\mathrm{M^+}$ and M.

2. Solvent / Ionic Liquid Properties

TABLE **S1**: Abbreviations of the solvents and ionic liquids (ILs) used in this study together with their relative dielectric constants (ε_r) at 20 °C (taken from the indicated references) and chemical structures.

solvent / ionic liquid	abbreviation	ε_r	chemical structure
tetrahydrofuran	THF	7.58[17]	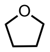
dichloromethane	DCM	8.93[17]	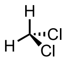
pyridine	PYR	12.91[17]	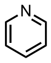
acetophenone	ACP	17.39[17]	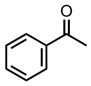
valeronitrile	VCN	19.71[17]	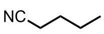
benzonitrile	BCN	25.2[17]	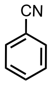
acetonitrile	ACN	35.94[17]	$NC-CH_3$
dimethyl sulfoxide	DMSO	46.45[17]	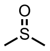
1-ethyl-3-methylimidazolium bis(trifluoromethylsulfonyl)imide	$[EtMeIm]^+[Tf_2N]^-$	12.3[18]	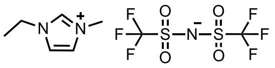
1-ethyl-3-methylimidazolium *n*-octylsulfate	$[EtMeIm]^+[n\text{oct-}SO_4]^-$	–	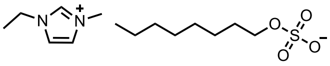
1-ethyl-3-methylimidazolium triflate	$[EtMeIm]^+[TfO]^-$	15.1[18]	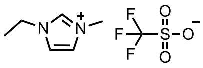
1-ethyl-3-methylimidazolium tetrafluoroborate	$[EtMeIm]^+[BF_4]^-$	12.9[18]	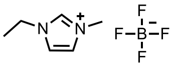
1-ethyl-3-methylimidazolium dicyanamide	$[EtMeIm]^+[N(CN)_2]^-$	11.0[19]	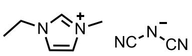
1-butyl-3-methylimidazolium thiocyanate	$[EtMeIm]^+[SCN]^-$	14.6[20]	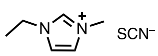
1-butyl-3-methylimidazolium dicyanamide	$[BuMeIm]^+[N(CN)_2]^-$	11.3[19]	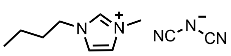

3. Quantum Chemical Calculations

All quantum chemical calculations were performed using Gaussian 16.[21] In order to compute reduction potentials (E^0_{A/A^-}) against Fc^+/Fc in accordance to Eq. 4 (main text), the following parameters need to be calculated:

- the ionization potential of Fc (IP_{Fc})
- the electron affinity of the analyte (EA_{EA})
- the solvation energy of Fc ($\Delta G^0_{\text{solv, Fc}}$)
- the solvation energy of Fc^+ ($\Delta G^0_{\text{solv, Fc}^+}$)
- the solvation energy of A ($\Delta G^0_{\text{solv, A}}$)
- the solvation energy of A^- ($\Delta G^0_{\text{solv, A}^-}$)

Ionization potentials and electron affinities were computed as the difference in the self-consistent field (SCF) energies of the fully optimized geometries of the respective redox pairs in vacuum, i.e.:

$$IP_{Fc} = E^{\text{vac.}}_{\text{opt, Fc}^+} - E^{\text{vac.}}_{\text{opt, Fc}} \tag{s22}$$

and:

$$EA_A = E^{\text{vac.}}_{\text{opt, A}} - E^{\text{vac.}}_{\text{opt, A}^-} \tag{s23}$$

The computed values therefore correspond to the adiabatic ionization potentials and electron affinities. Geometry optimizations were performed using the range-separated hybrid functional CAM-B3LYP in combination with the aug-cc-pVDZ basis set. This level of theory was chosen to more accurately describe the diffuse, loosely bound electrons in the anionic species, crucial for a more reliable computation of electron affinities.[22] It is known that conventional functionals, such as B3LYP, struggle with these diffuse species due to self-interaction errors.[22] We therefore opted to use the range-separated hybrid CAM-B3LYP functional[23] together with a basis set containing polarizable basis functions (aug-cc-pVDZ) to more properly describe the diffuse ions.[24] The electron affinities of the different molecules investigated in this study and the ionization potential of Fc calculated in this manner are summarized in Table **S2**.

TABLE **S2**: Calculated ionization potential (IP) as well as first (EA_1) and second (EA_2) electron affinities on the CAM-B3LYP/aug-cc-pVDZ level of theory for the indicated molecules.

molecule	IP / eV	EA_1 / eV	EA_2 / eV
Fc	7.140	-	-
TCB	-	2.680	-
TCNQ	-	3.805	−0.325
MV^{2+}	-	8.978	4.728

Solvation energies were calculated as the difference between the SCF energies of the fully optimized geometries of the respective species in vacuum and in solution, where the solvent environment was described using the polarizable continuum model (PCM).[25] Particularly, the solvation energy of species i was calculated according to:

$$\Delta G^0_{\text{solv,i}} = E^{\text{sol.}}_{\text{opt, i}} - E^{\text{vac.}}_{\text{opt, i}} \tag{s24}$$

The solvation energies of Fc, TCB, TCNQ, and MV^{2+}, along with those of their respective reduced species, which are needed for calculating the reduction potentials according to Eq. 4 (main text), are summarized in Tables **S3**-**S6**. Using these results together with the values listed in Table **S2**, Eq. 4 was used to compute the reduction potentials of the three compounds. The resulting calculated values are summarized in the tables in Section 5 and compared with the experimentally determined half-wave potentials from cyclic voltammetry.

TABLE **S3**: Calculated solvation energies of Fc and Fc^+ at the CAM-B3LYP/aug-cc-pVDZ level of theory employing a polarizable continuum solvation model.

solvent	ε_r [17]	$\Delta G^0_{solv,\,Fc}$ / eV	$\Delta G^0_{solv,\,Fc^+}$ / eV
THF	7.58	−0.085	−1.697
DCM	8.93	−0.089	−1.742
PYR	12.91	−0.095	−1.811
ACP	17.39	−0.099	−1.851
BZN	25.2	−0.103	−1.887
ACN	35.94	−0.105	−1.910
DMSO	46.45	−0.106	−1.923

TABLE **S4**: Calculated solvation energies of TCB and $TCB^{\bullet-}$ at the CAM-B3LYP/aug-cc-pVDZ level of theory employing a polarizable continuum solvation model.

solvent	ε_r [17]	$\Delta G^0_{solv,\,TCB}$ / eV	$\Delta G^0_{solv,\,TCB^{\bullet-}}$ / eV
THF	7.58	−0.480	−1.590
DCM	8.93	−0.501	−1.636
PYR	12.91	−0.534	−1.706
ACP	17.39	−0.554	−1.746
BZN	25.2	−0.573	−1.784
ACN	35.94	−0.585	−1.807
DMSO	46.45	−0.592	−1.821

TABLE **S5**: Calculated solvation energies of TCNQ, $TCNQ^{\bullet-}$ and $TCNQ^{2-}$ at the CAM-B3LYP/aug-cc-pVDZ level of theory employing a polarizable continuum solvation model.

solvent	ε_r [17]	$\Delta G^0_{solv,\,TCNQ}$ / eV	$\Delta G^0_{solv,\,TCNQ^{\bullet-}}$ / eV	$\Delta G^0_{solv,\,TCNQ^{2-}}$ / eV
THF	7.58	−0.426	−1.464	−5.349
DCM	8.93	−0.444	−1.505	−5.488
PYR	12.91	−0.473	−1.568	−5.703
ACP	17.39	−0.49	−1.604	−5.825
BZN	25.2	−0.507	−1.638	−5.938
ACN	35.94	−0.517	−1.658	−6.007
DMSO	46.45	−0.523	−1.671	−6.048

TABLE **S6**: Calculated solvation energies of MV^{2+}, $MV^{\bullet+}$ and MV at the CAM-B3LYP/aug-cc-pVDZ level of theory employing a polarizable continuum solvation model.

solvent	ε_r [17]	$\Delta G^0_{solv,\,MV^{2+}}$ / eV	$\Delta G^0_{solv,\,MV^{\bullet+}}$ / eV	$\Delta G^0_{solv,\,MV}$ / eV
THF	7.58	−5.763	−1.502	−0.212
DCM	8.93	−5.91	−1.541	−0.223
PYR	12.91	−6.136	−1.601	−0.239
ACP	17.39	−6.264	−1.636	−0.249
BCN	25.2	−6.382	−1.667	−0.259
ACN	35.94	−6.455	−1.687	−0.265
DMSO	46.45	−6.498	−1.699	−0.269

4. Additional Data

4.1 Tetracyanobenzene - TCB

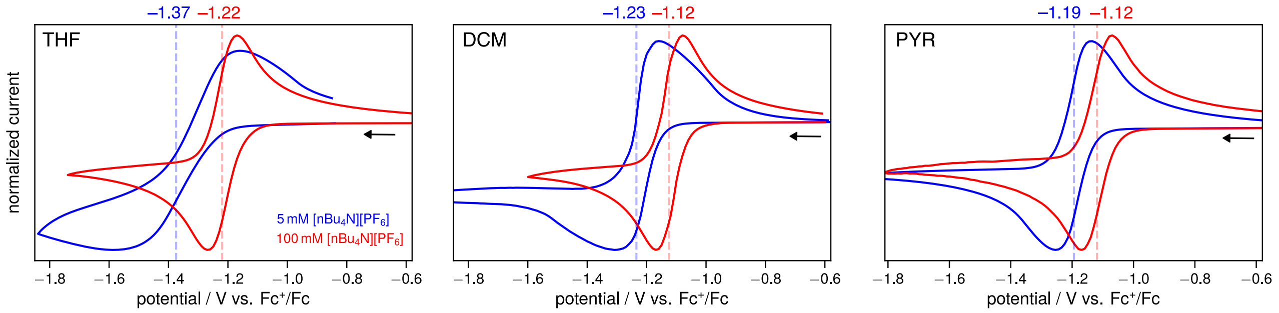

Figure S1: Normalized cylic voltammograms of the reduction TCB recorded at a glassy carbon working electrode internally referenced vs. Fc^+/Fc at a scan rate of 100 mV/s with either 100 mM (red) or 5 mM $[nBu_4N][PF_6]$ supporting electrolyte in THF (left), DCM (middle) and PYR (right). The half-wave potentials $E^2_{1/2} \approx E^0_{TCB/TCB^{\bullet-}}$) are shown as dashed lines.

TABLE **S7**: Experimental halfwave potentials ($E_{1/2}$) for the reduction of TCB in the investigated organic solvents and ionic liquids together with their bulk dielectric constants (ε_r) and theoretically computed standard reduction potentials ($E^{0,\text{theo.}}_{TCB/TCB^{\bullet-}}$) at the CAM-B3YLP/aug-cc-pVDZ/PCM level of theory versus Fc^+/Fc. a) 100 mM $[nBu_4N][PF_6]$, b) 5 mM $[nBu_4N][PF_6]$

solvent / ionic liquid	ε_r	$E_{1/2}$ / V	$E^{0,\text{theo.}}_{TCB/TCB^{\bullet-}}$ / V
THF	7.58[17]	-1.22^a / -1.37^b	−1.74
DCM	8.93[17]	-1.12^a / -1.23^b	−1.67
PYR	12.91[17]	-1.12^a / -1.19^b	−1.57
ACP	17.39[17]	-1.11^a	−1.52
VCN	19.71[17]	-1.10^a	-
BCN	25.2[17]	-1.12^a	−1.47
ACN	35.94[17]	-1.07^a	−1.43
DMSO	46.45[17]	-1.05^a	−1.41
$[EtMeIm]^+[Tf_2N]^-$	12.3[18]	−1.04	-
$[EtMeIm]^+[n\text{oct-}SO_4]^-$	-	−1.05	-
$[EtMeIm]^+[TfO]^-$	15.1[18]	−1.03	-
$[EtMeIm]^+[BF_4]^-$	12.9[18]	−1.00	-
$[EtMeIm]^+[N(CN)_2]^-$	11.0[19]	−1.03	-
$[BuMeIm]^+[N(CN)_2]^-$	11.3[19]	−1.06	-

4.2 Tetracyanoquinodimethane - TCNQ

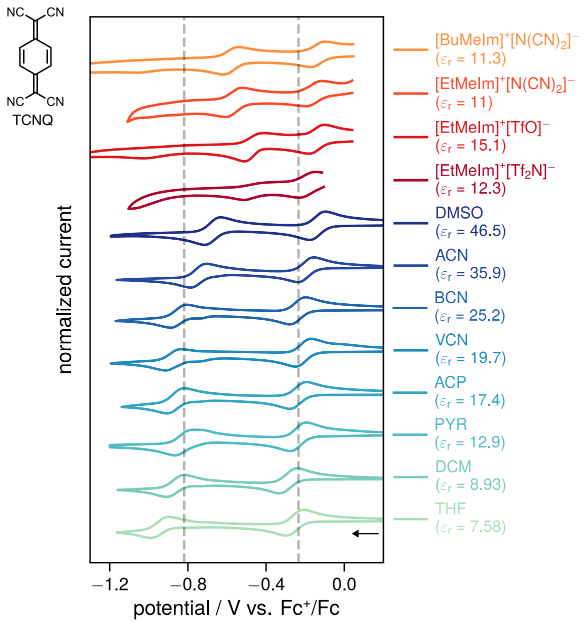

Figure S2: Normalized cylic voltammograms of the reduction of TNCQ in various organic solvents (blue shades) and ionic liquids (red shades) recorded at a glassy carbon working electrode internally referenced vs. Fc^+/Fc at a scan rate of 100 mV/s. For the organic solvents 100 mM of $[nBu_4N][PF_6]$ was added as supporting electrolyte. The dashed gray line correspond to the first ($E^1_{1/2} \approx E^0_{TCNQ/TCNQ^{\bullet-}}$) and second ($E^2_{1/2} \approx E^0_{TCNQ^{\bullet-}/TCNQ^{2-}}$) half-wave potentials in PYR to aid comparison.

TABLE **S8**: Experimental first ($E^1_{1/2}$) and second ($E^2_{1/2}$) half-wave potentials for the reduction of TCNQ in the investigated organic solvents and ionic liquids together with their bulk dielectric constants (ε_r) and theoretically computed first ($E^{0,theo.}_{TCNQ/TCNQ^{\bullet-}}$) and second ($E^{0,theo.}_{TCNQ^{\bullet-}/TCNQ^{2-}}$) standard reduction potentials at the CAM-B3YLP/aug-cc-pVDZ/PCM level of theory versus Fc^+/Fc. a) 100 mM $[nBu_4N][PF_6]$

solvent / ionic liquid	ε_r	$E^1_{1/2}$ / V	$E^2_{1/2}$ / V	$E^{0,theo.}_{TCNQ/TCNQ^{\bullet-}}$ / V	$E^{0,theo.}_{TCNQ^{\bullet-}/TCNQ^{2-}}$ / V
THF	7.58[17]	−0.25^a	−0.94^a	−0.69	−1.97
DCM	8.93[17]	−0.28^a	−0.86^a	−0.62	−1.83
PYR	12.91[17]	−0.23^a	−0.82^a	−0.52	−1.61
ACP	17.39[17]	−0.23^a	−0.86^a	−0.47	−1.49
VCN	19.71[17]	−0.21^a	−0.87^a	-	-
BCN	25.2[17]	−0.24^a	−0.85^a	−0.42	−1.38
ACN	35.94[17]	−0.19^a	−0.74^a	−0.39	−1.31
DMSO	46.45[17]	−0.13^a	−0.67^a	−0.37	−1.27
$[EtMeIm]^+[Tf_2N]^-$	12.3[18]	−0.19	−0.46	-	-
$[EtMeIm]^+[TfO]^-$	15.1[18]	−0.14	−0.46	-	-
$[EtMeIm]^+[N(CN)_2]^-$	11.0[19]	−0.16	−0.57	-	-
$[BuMeIm]^+[N(CN)_2]^-$	11.3[19]	−0.13	−0.58	-	-

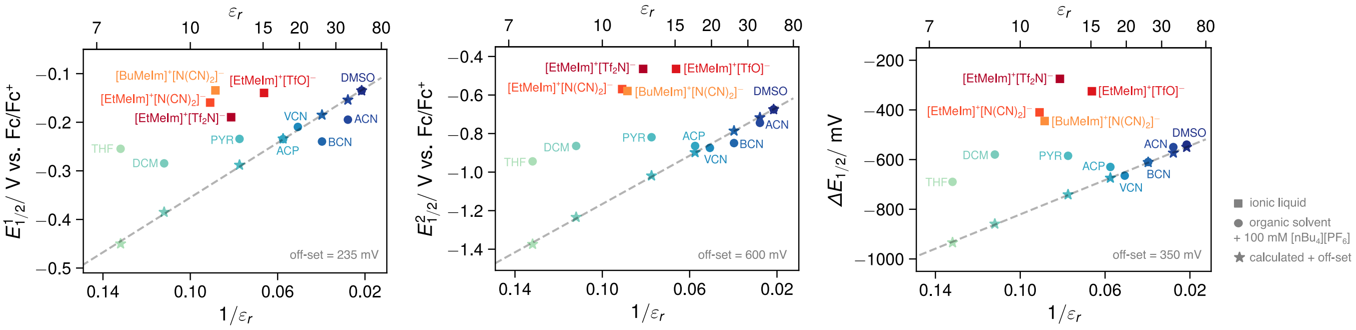

Figure S3: First ($E^1_{1/2} \approx E^0_{\mathrm{TCNQ/TCNQ^{\bullet-}}}$) and second ($E^2_{1/2} \approx E^0_{\mathrm{TCNQ^{\bullet-}/TCNQ^{2-}}}$) half-wave potentials as well as their difference ($\Delta E^2_{1/2} = E^2_{1/2} - E^1_{1/2}$) obtained from the CVs of the reduction of TCNQ in various organic solvents (blue shaded circles) and ionic liquids (red shaded squares) plotted against the inverse of their respective dielectric constant ($1/\varepsilon_r$). The stars represent calculated values from DFT calculations (off-set by the indicated amounts) employing a polarizable continuum model to calculate solvation energies in the organic solvents.

4.3 Methyl viologen - MV^{2+}

Applying Eq. 4 (main text) for the first and second reduction of MV^{2+} yields:

$$E^0_{\mathrm{MV^{2+}/MV^{\bullet+}}} = F^{-1}\left[\mathrm{EA_1} - \mathrm{IP_{Fc}} - \Delta\Delta G^0_{\mathrm{solv,\ MV^{\bullet+}/MV^{2+}}} - \Delta\Delta G^0_{\mathrm{solv,\ Fc^+/Fc}}\right] \tag{s25}$$

and

$$E^0_{\mathrm{MV^{\bullet+}/MV}} = F^{-1}\left[\mathrm{EA_2} - \mathrm{IP_{Fc}} - \Delta\Delta G^0_{\mathrm{solv,\ MV/MV^{\bullet+}}} - \Delta\Delta G^0_{\mathrm{solv,\ Fc^+/Fc}}\right] \tag{s26}$$

respectively. Assuming comparable solvation energies for Fc^+ and $MV^{\bullet+}$, i.e. $\Delta G^0_{\mathrm{solv,Fc^+}} \approx \Delta G^0_{\mathrm{solv,\ MV^{\bullet+}}}$, and applying the Born equation ($\Delta G^0_{\mathrm{solv,MV^{2+}}} \approx 4\Delta G^0_{\mathrm{solv,\ MV^{\bullet+}}}$), while neglecting solvation of the neutral species, all solvation terms in Eq. s25 simplify to $+2\Delta G^0_{\mathrm{solv,MV^{\bullet+}}}$. Since $\Delta G^0_{\mathrm{solv,MV^{\bullet+}}} < 0$ and becomes increasingly more negative with increasing solvent polarity, $E^0_{\mathrm{MV^{2+}/MV^{\bullet+}}}$ shifts to more negative values as polarity increases, i.e. the reduction of MV^{2+} becomes thermodynamically less favorable in more polar solvents, as the relatively larger solvation energy of MV^{2+} is lost upon reduction.

Using the same approximations in Eq. s26 leads to exact cancellation of the $\Delta G^0_{\mathrm{solv,Fc^+}}$ and $\Delta G^0_{\mathrm{solv,\ MV^{\bullet+}}}$ solvation terms and therefore, under these approximations, one would expect the second reduction potential $E^0_{\mathrm{MV^{\bullet+}/MV}}$ to be roughly independent on solvent polarity.

Subtraction of Eq. s26 from Eq. s25 shows that the standard potential separation $\Delta E^0_{1/2}$ can be written as:

$$\Delta E^0 = E^0_{\mathrm{MV^{\bullet+}/MV}} - E^0_{\mathrm{MV^{2+}/MV^{\bullet+}}} = F^{-1}\left[\mathrm{EA_2} - \mathrm{EA_1} - \Delta\Delta G^0_{\mathrm{solv,\ MV/MV^{\bullet+}}} + \Delta\Delta G^0_{\mathrm{solv,\ MV^{\bullet+}/MV^{2+}}}\right] \tag{s27}$$

Under the previous assumptions, the solvation terms simplify to $-2\Delta G^0_{\mathrm{solv,\ MV^{\bullet+}}}$ and hence $|\Delta E^0|$, i.e. the redox potential separation, is expected to become smaller as solvent polarity increases, similar to what is observed for TCNQ.

The experimental data (cf. Fig. **S4** / **S5**) nicely confirm these predicted shifts. Note that while the first half-wave potential shifts cathodically with increasing polarity, the shifts observed for the second reduction wave are much smaller in magnitude, as expected. In particular, the CVs in all measured ILs show a significantly cathodically shifted first reduction and a smaller half-wave potential separation compared to the measurements in PYR. The measurements in the ILs are instead comparable to those in polar solvents such as ACN or DMSO.

The quantum chemical calculations based on the continuum solvation model again qualitatively reproduce the observed trends in the organic solvents (cf. Fig. **S5**). The larger deviation of the PYR data point from the trend line likely arises from the preferential solvation effect of the supporting electrolyte salt in this moderately polar solvent. Note further, that the more sophisticated theoretical calculations predict a slight anodic shift of the second reduction potential with increasing

polarity, rather than a complete independence from it. The experimental data also show this behavior, with the second reduction peaks in the ILs being more anodically shifted than in PYR, and comparable in position to those observed in ACN and DMSO.

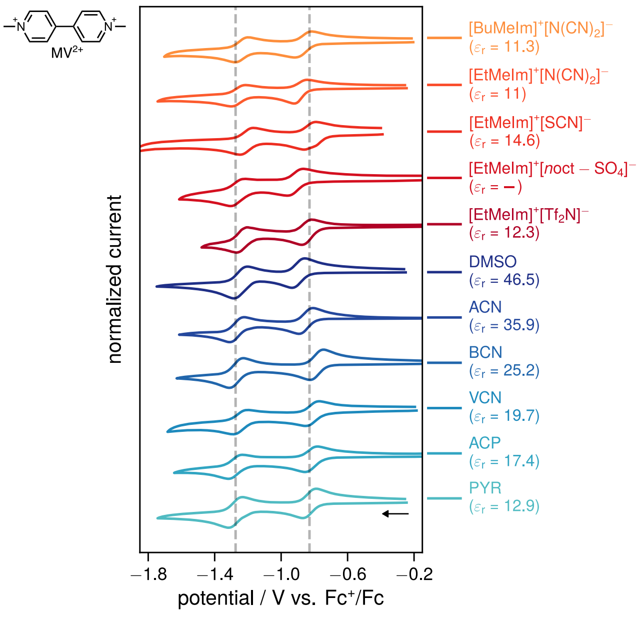

Figure S4: Normalized cylic voltammograms of the reduction of MV^{2+} in various organic solvents (blue shades) and ionic liquids (red shades) recorded at a glassy carbon working electrode internally referenced vs. Fc^+/Fc at a scan rate of 100 mV/s. For the organic solvents 100 mM of $[nBu_4N][PF_6]$ was added as supporting electrolyte. The dashed gray line correspond to the half-wave potentials for the first ($E^1_{1/2} \approx E^0_{MV^{2+}/MV^{\bullet+}}$) and second ($E^2_{1/2} \approx E^0_{MV^{\bullet+}/MV}$) reduction in PYR to aid comparison.

TABLE **S9**: Experimental first ($E^1_{1/2}$) and second ($E^2_{1/2}$) half-wave potentials for the reduction of MV^{2+} in the investigated organic solvents and ionic liquids together with their bulk dielectric constants (ε_r) and theoretically computed first ($E^{0,\text{theo.}}_{MV^{2+}/MV^{\bullet+}}$) and second ($E^{0,\text{theo.}}_{MV^{\bullet+}/MV}$) standard reduction potentials on the CAM-B3YLP/aug-cc-pVDZ/PCM level of theory versus Fc^+/Fc. a) 100 mM $[nBu_4N][PF_6]$

solvent / ionic liquid	ε_r	$E^1_{1/2}$ / V	$E^2_{1/2}$ / V	$E^{0,\text{theo.}}_{MV^{2+}/MV^{\bullet+}}$ / V	$E^{0,\text{theo.}}_{MV^{\bullet+}/MV}$ / V
THF	7.58[17]	0.00^a	0.00^a	−0.81	−2.09
DCM	8.93[17]	0.00^a	0.00^a	−0.88	−2.08
PYR	12.91[17]	−0.83^a	−1.27^a	−0.98	−2.06
ACP	17.39[17]	−0.82^a	−1.27^a	−1.04	−2.05
VCN	19.71[17]	−0.81^a	−1.24^a	-	-
BCN	25.2[17]	−0.79^a	−1.27^a	−1.09	−2.04
ACN	35.94[17]	−0.85^a	−1.27^a	−1.13	−2.03
DMSO	46.45[17]	−0.89^a	−1.24^a	−1.15	−2.03
$[EtMeIm]^+[Tf_2N]^-$	12.3[18]	−0.85	−1.23	-	-
$[EtMeIm]^+[n\text{oct}-SO_4]^-$	-	−0.91	−1.26	-	-
$[EtMeIm]^+[SCN]^-$	14.6[20]	−0.82	−1.21	-	-
$[EtMeIm]^+[N(CN)_2]^-$	11.0[19]	−0.88	−1.26	-	-
$[BuMeIm]^+[N(CN)_2]^-$	11.3[19]	−0.86	−1.24	-	-

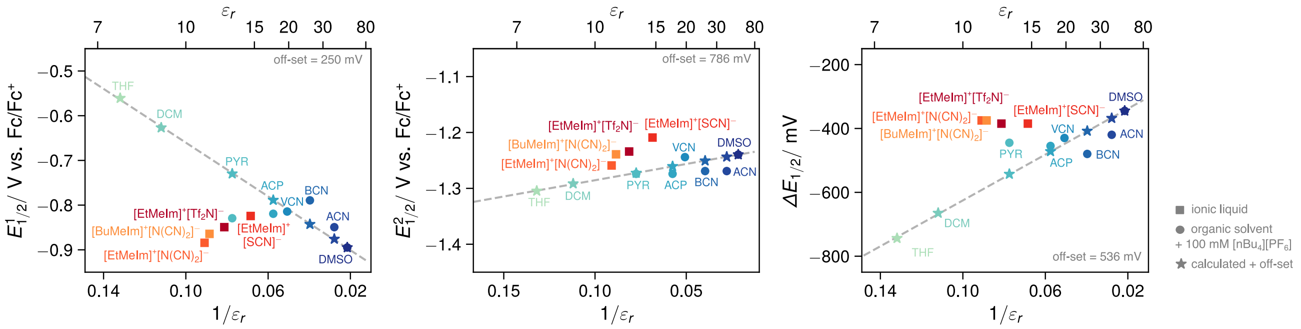

Figure S5: First ($E^1_{1/2} \approx E^0_{MV^{2+}/MV^{\bullet+}}$) and second (($E^2_{1/2} \approx E^0_{MV^{\bullet+}/MV}$) half-wave potentials as well as their difference ($\Delta E^2_{1/2} = E^2_{1/2} - E^1_{1/2}$) obtained from the CVs of the reduction of MV^{2+} in various organic solvents (blue shaded circles) and ionic liquids (red shaded squares) plotted against the inverse of their respective dielectric constant ($1/\varepsilon_r$). The stars represent calculated values from DFT calculations (off-set by the indicated amounts) employing a polarizable continuum model to calculate solvation energies in the organic solvents.

5. Control Experiments

In order to ensure that the half-wave potential shifts reported in this study are statistically robust, particularly in the highly viscous ionic liquids, we performed several control experiments to estimate the experimental uncertainty of the reported potentials. Figure **S6** shows ten consecutive CVs of the reduction of TCB in the ionic liquid $[EtMeIm]^+[N(CN)_2]^-$, recorded under the same experimental conditions used throughout this study (RE: Ag wire pseudo-reference, WE: glassy carbon, CE: Pt). The half-wave potential remains essentially independent of scan number and does not shift appreciably with time, varying by less than 10 mV over ten consecutive scans. Since the Fc^+/Fc internal standard is added and measured immediately after the first scan of the analyte measurement, while taking care not to disturb the electrode arrangement, this observation demonstrates that the pseudo-reference electrode potential remains stable over the course of the experiment. The resulting statistical uncertainty is well below the solvent-dependent potential shifts observed in this study (cf. Figs. 3–6), indicating that the reported shifts are significant and not attributable to experimental scatter, even in the viscous ILs.

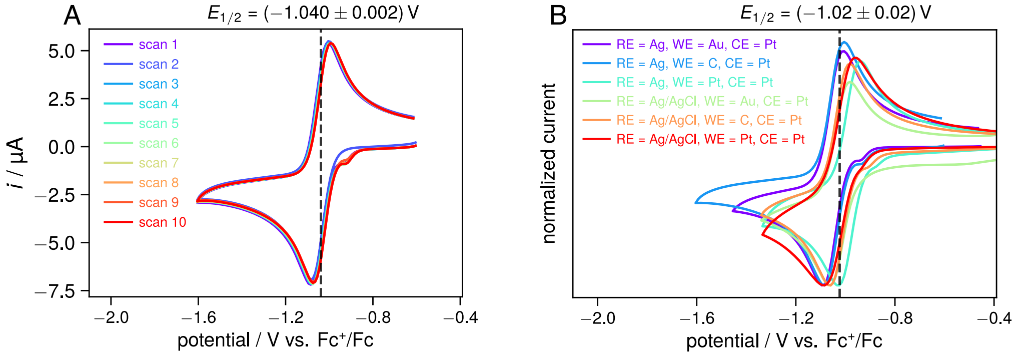

Figure S6: **A:** Cyclic voltammograms of TCB in $[EtMeIm]^+[N(CN)_2]^-$ recorded under the same experimental conditions used throughout this study (RE: Ag wire pseudo-reference, WE: glassy CE: Pt). Shown are ten consecutive scans which demonstrate the stability of the pseudo-reference electrode potential and, consequently, the invariance of the TCB reduction half-wave potential. **B:** Cyclic voltammograms of TCB in $[EtMeIm]^+[N(CN)_2]^-$ recorded either a pseudo-reference electrode or a fritted aqueous Ag/AgCl reference electrode, as well as different WE materials with the Pt CE.

Furthermore, electrochemical reduction potentials can be influenced by the structure of the electrode–electrolyte interface. In ILs in particular, pronounced charge ordering within the electric double layer is known to affect reaction energetics and overpotentials. To assess whether such interfacial effects contribute significantly to the observed half-wave potential shifts, we performed a series of control experiments probing the influence of the electrode material. As shown in Fig. **S6** B, CV of TCB in $[EtMeIm]^+[N(CN)_2]^-$ were recorded using either a pseudo-reference electrode (Ag wire) or a real fritted aqueous Ag/AgCl reference electrode, as well as different combinations of working electrode materials. While shifts in the extracted half-wave potentials are observed depending on the specific electrode configuration, the average values and their standard deviations lie within the uncertainty obtained using the electrode arrangement employed throughout the study

(cf. Fig. **S6** A). Accounting for variations due to electrode material and interfacial effects, we estimate a conservative upper bound of approximately 20 mV for the uncertainty in the reported half-wave potentials. This uncertainty is significantly smaller than the solvent-dependent shifts observed across different solvents and ILs (cf. Figs. 3–6 in the main text). We therefore conclude that, while interfacial effects may contribute at a minor level, they cannot account for the systematic and much larger potential shifts in the ILs reported here.

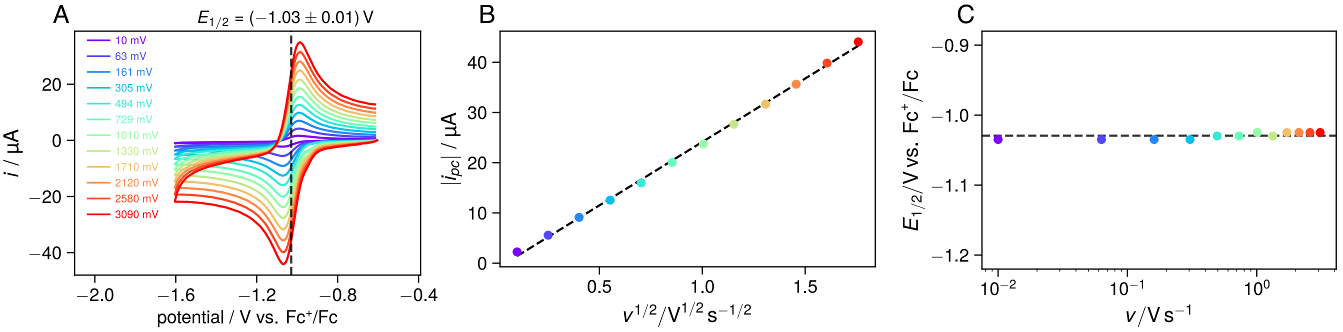

Figure S7: **A:** Scan-rate dependence of CVs of TCB in $[EtMeIm]^+[N(CN)_2]^-$ recorded under the same experimental conditions used throughout this study (RE: Ag-wire pseudo-reference, WE: glassy carbon, CE: Pt). **B:** Absolute value of the cathodic peak current as a function of $\sqrt{v}$, showing diffusion-controlled Randles-Ševčík behavior. **C:** Half-wave potential as a function of scan rate, demonstrating near invariance even in the viscous ionic liquid.

Finally, due to the high viscosity of the ILs, kinetic limitations or additional overpotentials could in principle influence the observed half-wave potentials. To assess this, we performed a scan-rate dependence study of the reduction of TCB in $[EtMeIm]^+[N(CN)_2]^-$, as shown in Fig. **S7**. The peak current is diffusion-controlled and follows the expected $\sqrt{v}$ dependence (cf. Fig **S7** B) according to the Randles–Ševčík equation. In addition, $E_{1/2}$ shows little to no dependence on scan rate (cf. Fig **S7** C), indicating that no significant additional overpotentials arising from mass-transport limitations are present, even in the viscous ILs.

6. References

[1] Born, M. Volumen und hydratationswärme der ionen. *Z. Angew. Phys.* **1920**, *1*, 45–48.
[2] Jackson, J. D. *Classical electrodynamics*; John Wiley & Sons, 2021.
[3] Debye, P.; Hückel, E. De la theorie des electrolytes. I. abaissement du point de congelation et phenomenes associes. *Physikalische Zeitschrift* **1923**, *24*, 185–206.
[4] McQuarrie, D. *Statistical Mechanics*; G - Reference,Information and Interdisciplinary Subjects Series; University Science Books, 2000.
[5] Case, B.; Hush, N.; Parsons, R.; Peover, M. The real solvation energies of hydrocarbon ions in acetonitrile and the surface potential of acetonitrile. *J. Electroanal. Chem.* **1965**, *10*, 360–370.
[6] Parker, V. D. Energetics of electrode reactions. II. The relationship between redox potentials, ionization potentials, electron affinities, and solvation energies of aromatic hydrocarbons. *J. Am. Chem. Soc.* **1976**, *98*, 98–103.
[7] Suppan, P. Medium effects in photoinduced electron transfer reactions. *Chimia* **1988**, *42*, 320–320.
[8] Shalev, H.; Evans, D. H. Solvation of anion radicals: gas-phase versus solution. *J. Am. Chem. Soc.* **1989**, *111*, 2667–2674.
[9] Bard, A. J.; Faulkner, L. R.; White, H. S. *Electrochemical methods: fundamentals and applications*, 2nd ed.; John Wiley & Sons, 2022.
[10] Savéant, J.-M.; Costentin, C. *Elements of molecular and biomolecular electrochemistry: an electrochemical approach to electron transfer chemistry*; John Wiley & Sons, 2019.
[11] Armarego, W. *Purification of Laboratory Chemicals: Part 1 Physical Techniques, Chemical Techniques, Organic Chemicals*; Butterworth-Heinemann, 2022.
[12] Sun, L.; Berglund, H.; Davydov, R.; Norrby, T.; Hammarström, L.; Korall, P.; Börje, A.; Philouze, C.; Berg, K.; Tran, A., et al. Binuclear ruthenium- manganese complexes as simple artificial models for Photosystem II in green plants. *J. Am. Chem. Soc.* **1997**, *119*, 6996–7004.
[13] Bott, A. Practical problems in voltammetry 3: reference electrodes for voltammetry. *Curr. Sep.* **1995**, *14*, 64–69.
[14] Inzelt, G.; Lewenstam, A.; Scholz, F. *Handbook of Reference Electrodes*; SpringerLink : Bücher; Springer Berlin Heidelberg, 2013.
[15] Elgrishi, N.; Rountree, K. J.; McCarthy, B. D.; Rountree, E. S.; Eisenhart, T. T.; Dempsey, J. L. A practical beginner's guide to cyclic voltammetry. *J. Chem. Educ.* **2018**, *95*, 197–206.
[16] Due to different solubilities of the analytes in the different solvents/ionic liquids it was difficult to keep the concentration the same among all solvents.
[17] Marcus, Y. *The Properties of Solvents*; The Properties of Solvents Bd. 1; Wiley, 1998.
[18] Weingärtner, H. The static dielectric constant of ionic liquids. *Z. Phys. Chem.* **2006**, *220*, 1395–1405.
[19] Hunger, J.; Stoppa, A.; Schrödle, S.; Hefter, G.; Buchner, R. Temperature dependence of the dielectric properties and dynamics of ionic liquids. *ChemPhysChem* **2009**, *10*, 723–733.

[20] Rybinska-Fryca, A.; Sosnowska, A.; Puzyn, T. Prediction of dielectric constant of ionic liquids. *J. Mol. Liq.* **2018**, *260*, 57–64.
[21] Frisch, M. J. et al. Gaussian~16 Revision C.01. 2016; Gaussian Inc. Wallingford CT.
[22] Jensen, F. *Introduction to computational chemistry*; John wiley & sons, 2017.
[23] Yanai, T.; Tew, D. P.; Handy, N. C. A new hybrid exchange–correlation functional using the Coulomb-attenuating method (CAM-B3LYP). *Chem. Phys. Lett.* **2004**, *393*, 51–57.
[24] Kendall, R. A.; Dunning Jr, T. H.; Harrison, R. J. Electron affinities of the first-row atoms revisited. Systematic basis sets and wave functions. *J. Chem. Phys.* **1992**, *96*, 6796–6806.
[25] Tomasi, J.; Mennucci, B.; Cammi, R. Quantum mechanical continuum solvation models. *Chem. Rev.* **2005**, *105*, 2999–3094.
